# Twenty-one years of three-way hybrid breeding shaped diversity and complementarity of male and female gene pools in sugar beet

**DOI:** 10.1007/s00122-026-05366-8

**Published:** 2026-09-19

**Authors:** Augustin Desprez, Karine Henry, Bruno Desprez, Pierre Devaux, Alain Charcosset, Maud I. Tenaillon, Laurence Moreau

**Affiliations:** 1 Université Paris-Saclay, INRAE, AgroParisTech, GQE - Le Moulon, 91190 Gif-sur-Yvette, France; 2 Florimond Desprez Veuve & Fils SAS, BP 41, 59242 Cappelle-en-Pevele, France; 3 Université Paris-Saclay, INRAE, CNRS, AgroParisTech, GQE - Le Moulon, EMR 9005, 91190 Gif-sur-Yvette, France; 4 United Beet Seeds (UBS), Industriepark 15, 3300 Tienen, Belgium

## Abstract

**Key message:**

Three-way hybrid breeding in sugar beet is associated with strong genomic differentiation between male and female parental pools and persistent haplotypic differentiation at agronomically relevant loci over the last two decades.

**Abstract:**

Three-way hybrid breeding has been used in several major crops to increase seed yield and manage heterosis. Sugar beet (*Beta vulgaris ssp. vulgaris*) breeding relies exclusively on it and combines cytoplasmic male sterility (CMS) and monogermity to exploit heterosis by combining male and female parental pools. The genomic consequences of this breeding scheme remain poorly characterized. We assembled a unique dataset comprising 1285 accessions spanning 21 years of selection within a breeding program, genotyped for 10,033 SNPs, to investigate genetic structuring and selection dynamics. Principal component and admixture analyses revealed a clear differentiation between parental pools. Over 21 years, males displayed a significant decline in within-pool diversity, accompanied by a rise in inter-pool differentiation. Linkage disequilibrium decreased in recent male lines, reflecting breeding practices that increase allele shuffling. Selection scans identified 174 outlier SNPs differentiating pools. Outliers clustered into seven haploblocks, including a region encompassing rhizomania-resistance gene (*Rz1*) and, more surprisingly, a region encompassing the domestication bolting locus B (*BvBTC1*). These results demonstrate that over the last 21 years, three-way hybrid breeding has increased differentiation between heterotic pools and shaped haplotypic variation at agronomically relevant loci, providing a genomic basis for future evaluation of their effects on agronomic performance.

**Supplementary Information:**

The online version contains supplementary material available at https://doi.org/10.1007/s00122-026-05366-8.

## Introduction

Beet domestication began over 10,000 years ago in present-day Turkey, with early selection focused on the leaves of the wild sea beet (*Beta vulgaris subsp. maritima*, thereafter *maritima*), later shifting toward root development, as evidenced by archeological remains in Egypt (~ 5500 years ago) and Greece (~ 2500 years ago). Various cultivated types subsequently diversified and spread across Europe during the Roman and Carolingian empires (Ulbrich 1934; Zohary 1988). Sugar beet currently covers around 16% of worldwide sugar production (FAO 2023) and has increased steadily from 6 million tons in 1900 (CEDUS 1993) to 42 million tons in 2023 (FAO 2023). Part of sugar beet’s success stems from its inherent ability to cope with the challenges of climate changes: Compared with other temperate crops, sugar beet is less vulnerable to dry summers and has inherited salt tolerance traits from its wild *maritima* ancestor (Skorupa et al. 2019).

At the genomic level, domestication of sugar beet caused a reduction in overall genetic diversity due to a demographic bottleneck and selective sweeps at key domestication loci, which reduced allelic diversity (Arnaud et al. 2009; Felkel et al. 2023). Despite this loss, domesticated populations display novel phenotypic variations absent in wild relatives (WR) (Wojcik et al. 2025), resulting from new allelic combinations and the selection of pre-existing mutations affecting yield, storage-root hypertrophy, bienniality (Pin et al. 2012; Frerichmann et al. 2013; Höft et al. 2018; Dhiman et al. 2025) and reduced dormancy. Beet wild relatives, particularly *maritima*, are genetically close to sugar beet and usually cross-compatible, allowing gene flow and introgression (Andersen et al. 2005; Galewski and McGrath 2020).

The cornerstone of sugar beet improvement has relied mainly on two simultaneous breakthrough discoveries made in the 1940s by USDA (United States Department of Agriculture). Cytoplasmic Male Sterility (CMS) (Owen 1945) enabled hybrid production in a species for which large-scale mechanical emasculation is unfeasible, and the genetic control of monogermity (production of fruits with a single seed) which avoids labor-intensive thinning at the seedling stage. Encoded by a single recessive allele (*m*), the latter greatly simplified crop management and became widely adopted from the 1950s onward. Using both discoveries in breeding raised a dual challenge: (i) the establishment of breeding methods allowing the production of hybrid varieties from a male sterile (female) pool and a fertile male pool, and (ii) the development of high-performing monogerm varieties, in which at least the female parent producing the variety seeds has to be homozygous for the recessive *m* allele conferring monogermity.

**Fig. 1:**
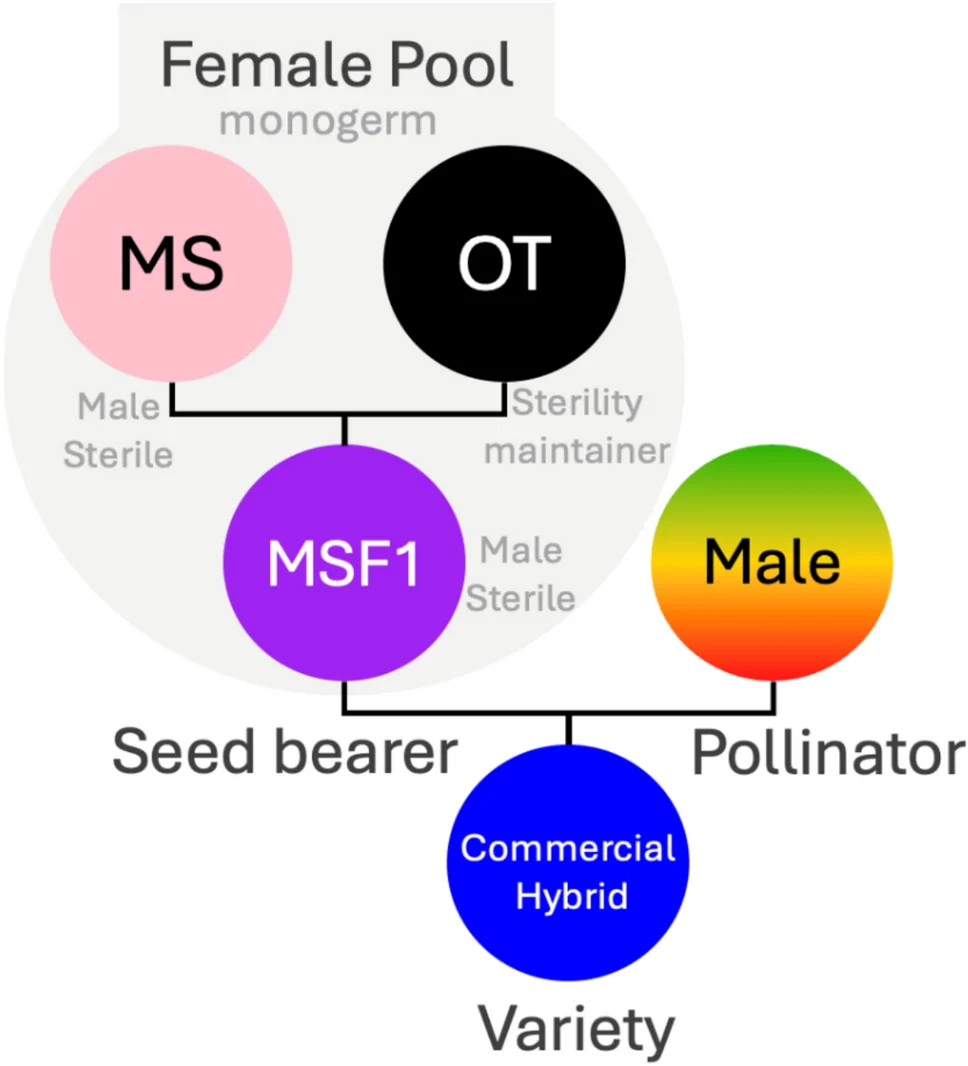
Three-way hybrid breeding scheme. The two parental pools, female (MS, OT, and the MSF1 seed bearer) and male (Pollinator), and the variety pool (commercial three-way hybrids) are shown

Strong inbreeding depression inherited from self-sterility in wild beets leads to significant yield and seed production losses, making inbreeding a major obstacle in hybrid breeding programs. Hence, an inbred monogerm seed parent typically suffers from reduced vigor and fertility (seed yield). To circumvent this limitation, Hogaboam established in 1957 the first populations of male sterile (MS) lines, O-Type (OT) maintainers (named after Dr. F.V. Owen), and males (Owen 1945; Hogaboam 1957). Crossing MS with OT produces MSF1 (first-generation hybrid male sterile) seed parents (females) with sufficient vigor to be crossed with pollinators (males) to produce commercial hybrid varieties (Fig. 1). This three-way hybrid method enabled the production of relatively uniform hybrid varieties while overcoming inbreeding depression when fixing monogermy in the female parent.

Three-way hybrid breeding scheme results in an asymmetrical breeding between male and female pools. Specifically, there are two generations between the female parents and the evaluated hybrids, whereas a single generation separates the male pollinators from evaluated hybrids. Over 60 years of breeding within male and female pools aimed at enhancing the complementarity between them by improving their General Combining Ability (GCA) for traits related to sugar yield (Labroo et al. 2021). This process is expected to have led to the differential fixation of alleles between parental pools due to selection or drift (Adetunji et al. 2014) as observed in maize after switching to hybrid breeding (Comstock et al. 1949; Menz Rademacher et al. 1999; Hinze et al. 2005; Edwards 2011). Along with a selection for GCA for yield-related traits, sugar beet breeders have used genetic resources such as WRs, public accessions, or varieties to introduce new traits. Those include, for example, two dominant rhizomania tolerances: *Rz1* from the Holly Sugar Company germplasm and *Rz2* from WRs (Biancardi et al. 2002; Capistrano-Gossmann et al. 2017). Another valuable trait introduced is beet cyst nematode (BCN) tolerance from accession WB242 in the early 2000s (Scholten and Lange 2000; Panella et al. 2020). These introductions may also have impacted diversity patterns along the genome.

The objective of this article is to document the main genomic consequences of forces at stake in a context combining both reciprocal recurrent selection breeding and trait introductions. Relying on the genotyping of over 11,000 plants and the recent reference genome (McGrath et al. 2023), this study leverages a uniquely comprehensive dataset, encompassing twenty-one years of selection within the United Beet Seed breeding program—an unprecedented resource for examining genetic structuring, selection dynamics and complementarity between parental pools. Our objectives were to (1) investigate the genetic impact of the structuring of the breeding material into heterotic pools, (2) evaluate genetic diversity within—and differentiation between pools and their evolution through time, and (3) identify genomic regions showing strong differentiation between parental pools and potentially associated with selection.

## Methods

### Plant material and genotyping data

A total of 11,099 plants from the germplasm of the United Beet Seed company were grown and genotyped between 2015 and 2023. They represent 1285 accessions including 299 females (23 MS, 144 OT and 132 MSF1), 605 males (pollinators) and 381 three-way hybrids (potential commercial varieties). Because accessions show varying levels of genetic heterogeneity, we analyzed from six to 12 plants per accession. The breeding time of each accession was determined as the year of the initial cross from which it derived, before undergoing two to four generations of self-pollination.

We genotyped all plants using 13,005 single-nucleotide polymorphisms (SNPs) from the United Beet Seed-designed 35 K Axiom® genotyping chip from Affymetrix (CA, USA). Briefly, the chip was developed based on SNPs discovered from a collection of 16 individuals chosen from over 10,000 accessions including fodder beet (*Beta vulgaris* ssp. *vulgaris*) and wild beet (*Beta vulgaris* ssp. *maritima*) (Mangin et al. 2015; Pegot-Espagnet et al. 2019). SNPs were selected based on their relevance, genotyping reliability and uniform distribution across the genome (Huyghe et al. 2020; Dhiman et al. 2024). After filtering for biallelic SNPs, we obtained 10,033 SNPs positioned on the nine pseudochromosomes (nine largest scaffolds) of the reference genome of EL10.2. Genotyping data were curated to eliminate potential labeling errors and pollen contamination by removing off-type genotypes, identified using Manhattan distances between individuals within each accession (Supplementary Information: Genotyping data management).

### Genotyping data processing

Each accession represented either the progeny obtained from self-fertilization of a single individual (S2 or S3 lines for the OT, MS and male accessions) or several individuals in the case of hybrid accessions (MSF1 hybrids or three-way hybrids). Using genotypic data from multiple plants (N) per accession, we determined a single consensus genotype.

First, for a marker with two alleles A and B, we defined six possible genotypic profiles for the accession based on the expected genotypic distribution of its offspring—100% of offspring AA corresponding to an accession genotypic profile [AA], 100% BB [BB], 100% AB [ABfix], 50% AB and 50% AA [AAB], 50% AB and 50% BB [ABB], 50% AB, 25% AA and 25% BB [ABseg] (Supplementary Information, Supplementary Table S1). Second, for each accession we computed the likelihood associated with each genotypic profile based on the observed numbers of individual plants carrying genotypes AA BB and AB at each marker (n_AA_, n_BB_ and n_AB_), and an error rate R, accounting for genotyping errors (3–5%) (Montanari et al. 2019), considering the mutation rate to be negligible (1.3 × 10^–8^ per base pair per generation, McGrath et al. 2023).

For example, the likelihood of [AA] profile for a given accession was computed as follows:

$L\left(AA | ({n}_{\mathrm{A}\mathrm{A}},{n}_{\mathrm{B}\mathrm{B}},{n}_{\mathrm{A}\mathrm{B}}) \right)=\frac{{\left(1-R\right)}^{{n}_{\mathrm{A}\mathrm{A}}}{\left(\frac{-1+\sqrt{1+4R}}{2}\right)}^{{n}_{\mathrm{A}\mathrm{B}}}{{\left(\frac{-1+\sqrt{1+4R}}{2}\right)}^{2}}^{{n}_{\mathrm{B}\mathrm{B}}}N!}{{n}_{\mathrm{A}\mathrm{A}}! {n}_{\mathrm{A}\mathrm{B}}! {n}_{\mathrm{B}\mathrm{B}}!}$ with similar trinomial equations for other genotypic profiles (Supplementary Information S1, Supplementary Table S2).

If the ratio of the most likely accession genotypic profile exceeded that of the second most likely by a factor Q set to 2, we attributed the former as the consensus genotypic profile. Otherwise, it was considered missing (NA). The values of R (= 0.04) and Q (= 2) were determined from a subset of accessions genotyped on a large number of plants, for which it was possible to establish a reliable consensus genotype and estimate the error rate. Three examples of the consensus genotype procedure alongside a schematic representation are provided in Supplementary Information.

Since the standard variant calling format (VCF) supports only three genotype categories, genotypic profiles displaying both alleles—[ABfix], [AAB], [ABB] and [ABseg]—were grouped into a single profile [AB], alongside the two homozygotes [AA] and [BB]. We performed all subsequent analyses on the resulting consensus genotypes transformed into VCF format without filtering for minor allele frequency.

### Genetic structure and ancestry inference

A principal component analysis (PCA) was performed on the complete set of 10,033 SNPs. No LD pruning was applied prior to PCA because the reduced set of markers used in the chip was selected to provide well-distributed marker positions (Supplementary Figure S2). Given that LD can influence PCA, the results were interpreted together with ADMIXTURE and pairwise differentiation analyses rather than as standalone evidence of population structure. The first five principal components (PCs) were plotted for visual examination of the genetic structure using R packages FactoMineR and FactoExtra (Lê et al. 2008).

We inferred global ancestry by using an unsupervised admixture maximum likelihood approach implemented in ADMIXTURE (v1.3.0, Alexander et al. 2009) considering different numbers of ancestral clusters, *K* (*K* = 2, 3, 4, 5, 6, 8, 10, 20, 30, 40, 50 or 60). We determined the optimal number of clusters (*K*) using cross-validation (CV). Because cross-validation values did not identify a clear optimal K, ADMIXTURE was used as an exploratory description of hierarchical structure rather than as a formal inference of the true number of ancestral populations.

### Patterns of diversity, linkage disequilibrium and differentiation

Genetic diversity was measured using the mean of modified Rogers’ distance (MRD) (Wright 1978; Goodman and Stuber 1983) between pairs of individuals within breeding pools considering three seven-year eras in pollinators. MRD was computed as:

$${\mathrm{MRD}} = \frac{1}{{\sqrt {2m} }}\sqrt {\mathop \sum \limits_{i = 1}^{m} \mathop \sum \limits_{j = 1}^{2} \left( {p_{ij} - q_{ij} } \right)^{2} }$$

where *m* is the number of loci and *p*_*ij*_ and *q*_*ij*_ the frequencies of allele j at locus i for the two individuals, respectively. The number of alleles at locus *i* is 2, as only biallelic markers were kept.

Linkage disequilibrium (LD) within breeding pools was estimated between SNPs with a MAF  >  1% across all nine chromosomes. We used the usual *r*^2^ estimate of correlation between pairs of SNPs genotypes computed with the LDcorSV R package (v1.3.1, Mangin et al. 2012). LD landscape within chromosomes of each pool was explored by plotting the mean *r*^2^ between markers within a 500-kb sliding window and a 100-kb step.

We measured differentiation between parental pools using the estimate of *Fst* from Weir and Cockerham (Weir and Cockerham 1984; Weir and Hill 2002), computed with vcftools (v0.1.16, Danecek et al. 2011). This estimate is robust to differences in population sample sizes, unlike the Hudson’s Fst (Hudson et al. 1992), which can be inflated when population sizes are highly unbalanced such as in our dataset. Fst between the female hybrids (MSF1) and the male parental pools was calculated across  three seven-years eras, treating the female hybrid pool as constant over time.

### Selection scans

We identified outlier SNPs differentiating the male and MSF1 heterotic pools using two complementary approaches. First, we computed per-marker Fst values (Weir and Cockerham 1984) and selected outlier SNPs exceeding the 95th percentile of the genome-wide distribution, corresponding to a threshold of Fst >  0.77 (n = 470 SNPs).

We complemented this threshold-based approach with a second model-based method using the PCA-based approach implemented in PCAdapt (Luu et al. 2017), in which z-scores were calculated from the first principal component, the one distinguishing male from female pools. PCAdapt identified outlier SNPs associated with pool differentiation at a false discovery rate (FDR, Benjamini and Hochberg 1995) threshold of 10%. The intersection between the Fst and PCAdapt outlier sets was used to define a list of highly differentiated regions, corresponding to candidate regions differentially selected between pools.

Furthermore, we identified temporal outlier SNPs in the male pool, i.e., SNPs that showed extreme temporal changes in allele frequencies across the three eras. In order to do so, we used the standardized variance in allele frequencies (Fc) from Nei and Tajima (Nei and Tajima 1981) implemented in the method from Goldringer and Bataillon (2004). We computed Fc across loci between the first and third breeding eras, we fitted them to a beta distribution and identified loci with extreme Fc-values compared to the genome-wide Fc using a false positive rate of 5%.

### Haploblocks and haplotype networks

We used data from all the parental pools to construct haploblocks with the Solid Spine of LD (SPI) method, as described by the developers of Haploview (Barrett et al. 2005). Briefly, this method defines the boundaries of an LD block by extending a block of one SNP to the next as long as the pairwise *D*’ value between the two most distant SNPs exceeds a specific threshold (set to 0.2). We used the resulting haploblocks to study the corresponding networks of haplotypes within a haploblock for genomic regions showing elevated differentiation, i.e., defined as a cluster of outlier SNPs. In addition, we examined the haploblock structure in the chromosome 5 region associated with BCN tolerance which provides a well-characterized example of recent selection. The tolerance allele was introgressed from WR into a single parental pool, the male one, in the early 2000s and subsequently selected in a subset of the breeding germplasm.

In order to identify haplotypes within haploblocks, we phased the genotypic data and imputed missing data using Beagle (v5.5, Browning et al. 2021) with the effective population size (N_e_) set to 4, window size set to 100  cM, burn-in set to 90, iterations to 5000 and phases states to 30. All parameters were adjusted in a previous publication to mimic recombination patterns in inbred breeding material with a small founder population while accounting for accuracy and efficiency of imputation and phasing (Niehoff et al. 2022). All other parameters were set to default values. MS and OT were phased jointly and were used as a reference panel for MSF1 phasing. Males were phased separately.

The effective number of haplotypes (HAe) per block and pool was computed using.

${\mathrm{HAe}} = \frac{1}{{\mathop \sum \nolimits_{{\text{i = 1}}}^{{\mathrm{H}}} {\mathrm{p}}_{{\mathrm{i}}}^{2} }}$ with H being the total number of distinct haplotypes in the pool and p_i_ the frequency of the i^th^ haplotype. Haplotype networks were computed on top outlier haploblocks using the R package Pegas (Paradis 2010) which utilizes a Hamming distance between haplotypes to construct a parsimony network.

### Gene annotation and candidates

Genes within regions linked to outlier or temporal outlier SNPs were identified using the EL10 reference genome annotation (McGrath et al. 2023). The EL10 assembly was annotated using the MAKER pipeline with two gene prediction programs AUGUSTUS and SNAP that were trained using the StringTie transcript sequences.

Among all annotated genes, we focused on those with known or predicted effects on traits under breeding-related selection (root weight yield, sugar content, disease resistances, grain yield).

## Results

### Heterotic pools and breeding eras shape genetic structure

**Fig. 2:**
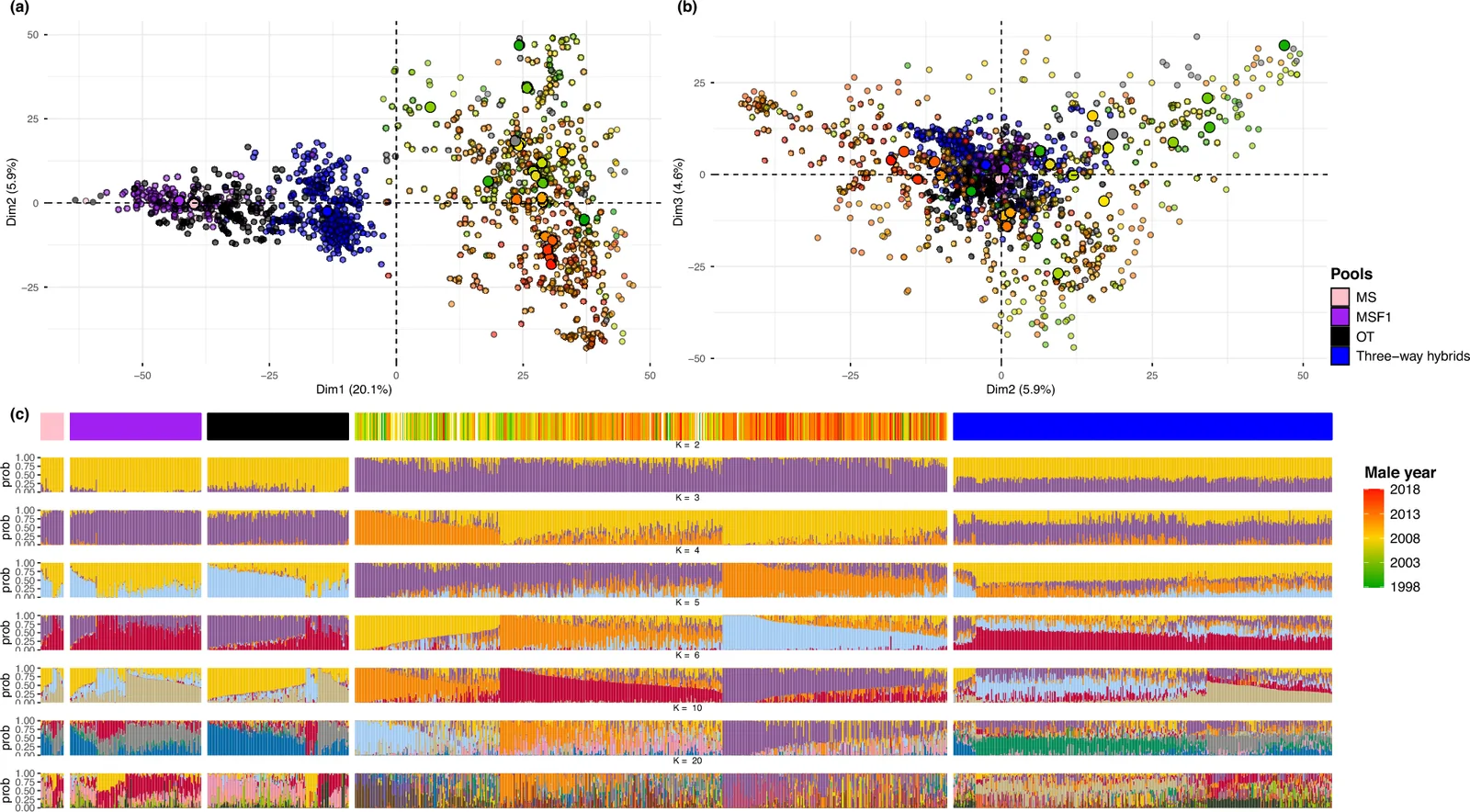
Genetic structure of the breeding panel. PCA computed on 10k SNPs genotyped on 1285 accessions (dots) with PC1 and PC2 (**a**) and PC2 and PC3 (**b**) shown with colors corresponding to breeding pools (MS: pink, OT: black, MSF1: purple, Hybrids: blue, males displayed with a gradient from older to more recent accessions). Gray dots correspond to males with unknown years. Large dots are the centroids (values averaged by pool and by year for the male pool). ADMIXTURE analysis of 1285 beet accessions for *K* = {2,3,4,5,6,10,20} (**c**). Each beet accession is represented as a vertical bar segmented into K distinct colors, indicating the estimated ancestry proportion of each cluster. The first-row colors correspond to the known structure of the panel: 23 MS in pink, 144 OT in black; 132 MSF1 in purple, 381 commercial three-way hybrids in blue; 605 males in a gradient from older (green) to more recent (red) accessions, white for unknown years

The first axis of the Principal Component analysis (PC1), which explained 20.1% of the variation, highlighted that the main driver of diversity structuring was the differentiation between the male and MSF1 pools, with three-way hybrid varieties occupying an intermediate position (Fig. 2a). Within the female pool, PC1 also revealed a clear genetic structure with a marked differentiation between MS and OT. PC2, which explained 6% of the variation captured differentiation within the male pool. This structure was strongly associated with breeding time as evidenced by the color gradient of centroids ranging from green to red along PC2, from the oldest to the most recent males (Fig. 2b).

The ADMIXTURE analysis revealed no optimal number of ancestral clusters (*K*) based on the cross-validation method. Cross-validation values decreased continuously from 0.6 at *K *= 2 to 0.4 at *K *= 20 (Supplementary Figure S3). We therefore did not interpret any single K as the true number of ancestral populations. Instead, ADMIXTURE was used as an exploratory description of hierarchical structure. We focused on *K* = 2, *K *= 3 and *K* = 5 because these values captured relevant patterns (Fig. 2c): first the separation of male and female pools at *K* = 2, with three-way hybrid varieties being admixed between both pools; second the differentiation of the oldest male accessions at *K *= 3; and finally, the subdivision of male accessions into three eras starting at *K* = 5. The three eras (1998–2004, 2005–2011, and 2012–2018) spanned 21 years of selection, and comprised 93, 161, and 342 males, respectively (nine males have unknown years). In contrast to males, we detected no time-related structure in the female pools.

### Male selection: declining diversity and rising differentiation between heterotic pools

**Fig. 3:**
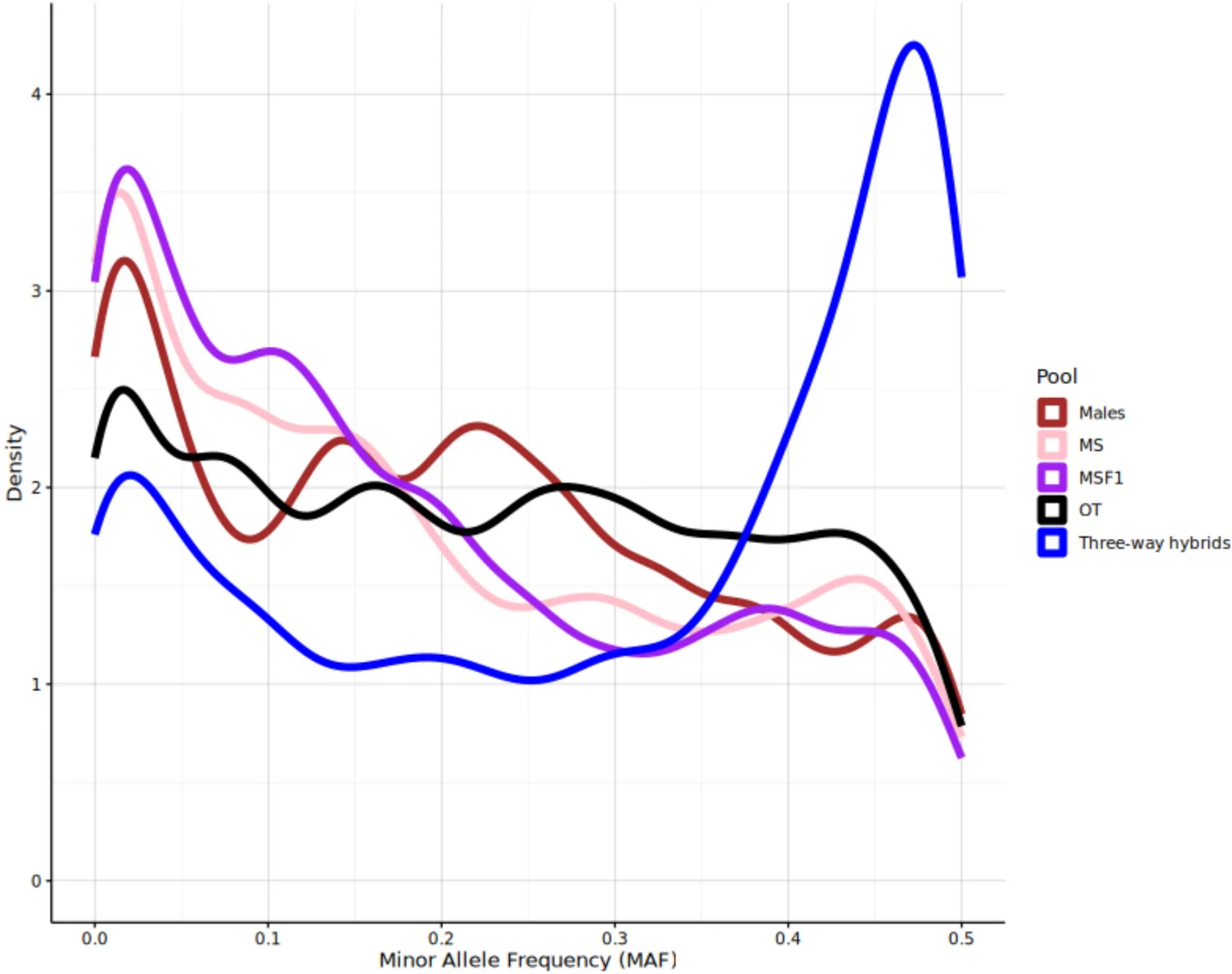
Comparison of the MAF density per pool. Commercial three-way hybrids: blue; Male parents in brown; Female parents: MS in pink, OT in black

We investigated the allele frequencies within the parental pools. Males, MS and OT displayed similar spectra with a deficit of low-frequency alleles, in line with what is expected from ascertainment biases in SNP arrays (Fig. 3). The proportion of markers with a MAF over 45% strikingly differed between groups. It corresponded to 8% of the markers in the male, MS and OT pools but 29% of the markers in three-way hybrids. This difference is due to a large proportion of markers being heterozygous in the three-way hybrid varieties, that are obtained from crosses between accessions from the male and female heterotic pools.

**Fig. 4:**
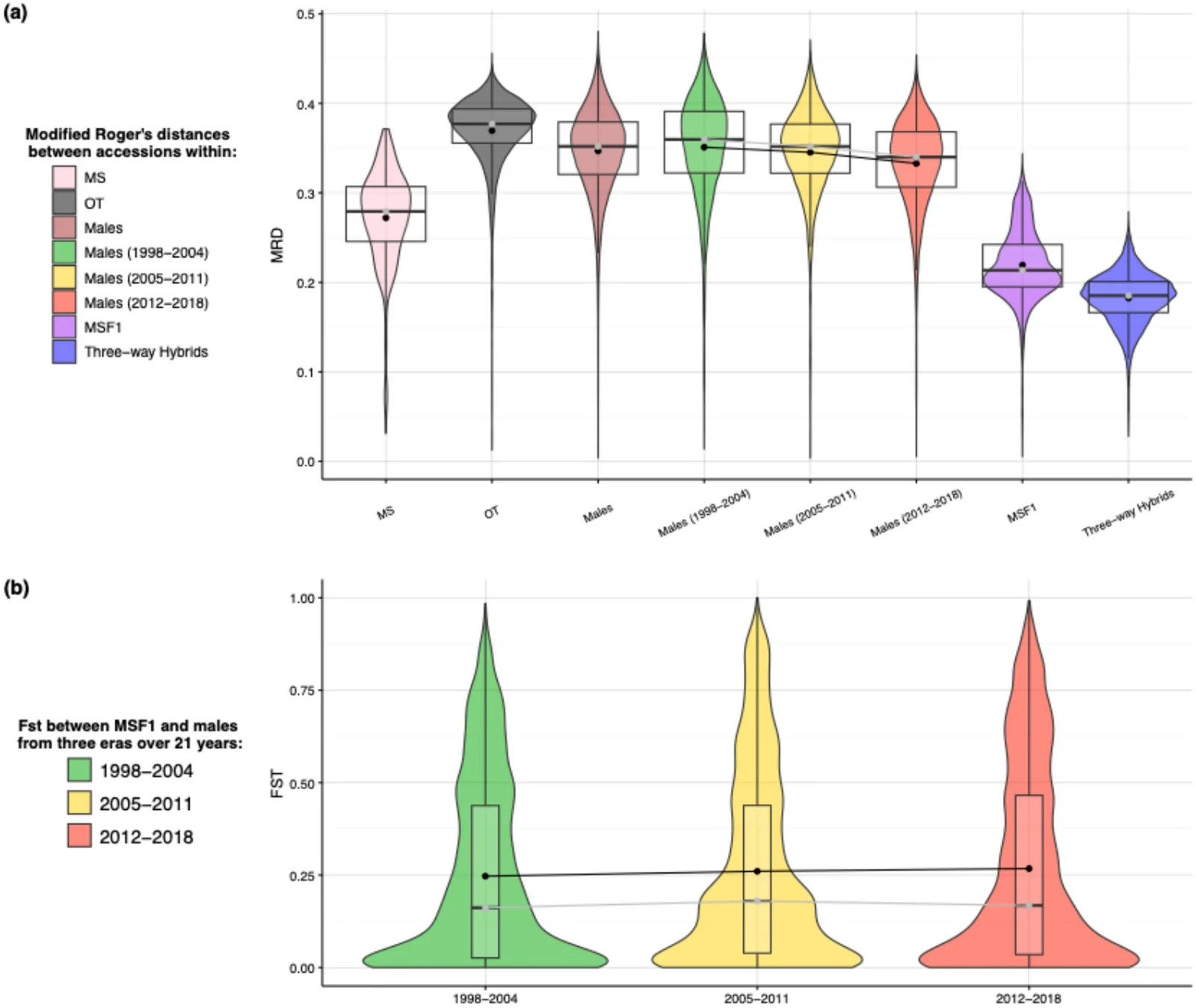
Diversity and differentiation in the breeding panel. Genetic diversity as measured by MRD within each pool (MS, OT, Males, MSF1 and hybrids) and by breeding era for the male pool (**a**) and differentiation as measured by the distribution of Fst between males and MSF1 by breeding era (**b**). Medians are shown in gray and means in black

To assess how genetic diversity has evolved within and between breeding pools over time, we analyzed patterns of allelic variation using the modified Rogers’ distance (MRD) and Fst estimates. Considering all three breeding eras together for males, genetic diversity levels within males (0.35) and OT (0.37) were similar, while MS (0.27) displayed lower diversity (Fig. 4a). Males displayed greater MRD variance than the MS female pool (Supplementary Figure S4) as expected from the genetic structuring revealed by breeding eras (Fig. 2c). Considering those eras separately, the diversity measured by MRD within males displayed a slight yet significant decrease over time (*t*-test, *p*-values < 0.0001) from 0.35 (ERA 1) to 0.34 (ERA 2) and to 0.33 (ERA 3). This result is in line both with the PCA highlighting a divergence along a historical gradient (Fig. 2b) and the admixture results showing that subgroups emerged within males with increasing *K* values (Fig. 2c). MRD within hybrids (MSF1 and three-way hybrids) was lower than within parental pools, which can be explained by the high proportion of heterozygous genotypes in the hybrid accessions.

We computed the Fst as a measure of allele differentiation between males and female hybrids (MSF1 obtained from crosses between MS and OT), to evaluate the genomic differentiation between the male and female pools used as parents of three-way hybrids. Because the female pool did not contain enough accessions with sufficiently balanced temporal representation, MSF1 accessions were treated as a single reference group across male breeding eras. Therefore, temporal changes in Fst should be interpreted as changes in the male pool relative to the sampled female hybrid pool, rather than as a direct estimate of simultaneous divergence in both parental pools. The distribution of Fst across all markers revealed that most markers had similar allele frequencies in the male and female pools, resulting in a skewed distribution with low Fst values and a long tail extending toward higher values (Fig. 4b). The predominance of low Fst values reflected the recent common origin of both pools (60 years since sugar beet hybrid development). Across breeding eras, Fst displayed a subtle but steady increase from 0.25 to 0.27. In parallel, highly differentiated markers accumulated over time, as indicated by an extension of the upper tail of the Fst distribution and an increasing proportion of markers with Fst > 0.5 (Fig. 4b). This proportion increased from 19.3% (1998–2004), to 20.1% (2005–2011) and 22.7% (2012–2018), representing a relative increase of 18%. Overall, these results indicate a modest increase in genomic differentiation between the male pool and the sampled MSF1 reference over the course of the 21-year breeding period.

### Correlated patterns of diversity and differentiation across the genome

**Fig. 5:**
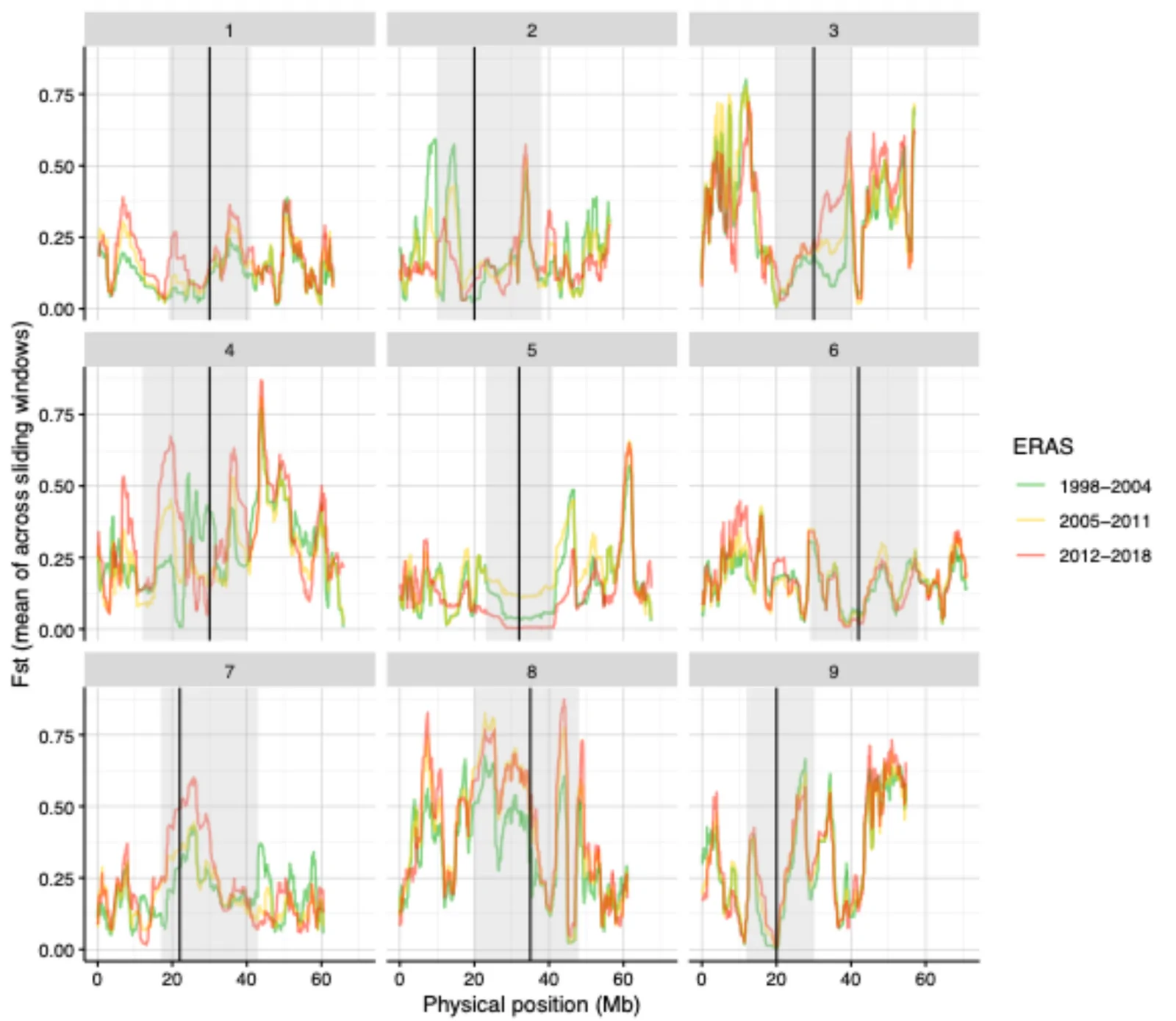
Genome-wide Fst between male pool and MSF1 per breeding era. Pericentromeric regions are shown with shaded areas with centromeres as vertical black lines

Chromosome-by-chromosome analysis of the Fst between pollinator and MSF1 revealed distinct patterns with average values across breeding eras ranging from 0.15 for chromosome 1 to 0.37 for chromosome 8. Notably, chromosomes 3, 4, 8, and 9 exhibited higher Fst values, with a tendency of increased differentiation between the first and the last era. As illustrated on chromosome 8, the average Fst between the first and third era increased by 22% while the median increased by only 5%. This pattern was reflected in the mean rather than the median because Fst rose primarily in genomic regions that were already highly differentiated (Fig. 5, Supplementary Figure S5). Chromosome 3 revealed a reverse pattern with a rather stable mean Fst (+ 6%) but an increased median Fst (+ 44%) over time (Fig. 5). Chromosomes 1, 2, 5, 6, and 7 consistently displayed lower Fst values than the other chromosomes (Supplementary Figure S5), indicative of a greater genetic similarity between male and female hybrid pools. Substantial differences were also observed among genomic regions within chromosomes, such as on chromosome 2 and 5 where some regions exhibited an increase and others a decrease of Fst over time (Fig. 5). Variations in sizes of pericentromeric genomic regions may cause these patterns, as observed in chromosomes 5 (Fig. 5) with a corresponding 10-Mb-wide plateau whose Fst value decreased across eras.

Interestingly, the observed patterns of within-pool diversity (MRD) and between-pool differentiation (Fst) appeared linked with a significant Pearson's correlation coefficient of − 0.67 (*p*-value = 10^–4^) between eras. Chromosome 4, for example, exhibited a sharp decrease in diversity through time and also displayed Fst increase, particularly between the two most recent eras (Supplementary Figure S5a). Such pattern, also observed on chromosomes 3, 8 and 9 reflected an increased genetic differentiation between the male pool and the female hybrid pool at the expense of within-male diversity.

### LD decreased over breeding eras in the male heterotic group

**Fig. 6:**
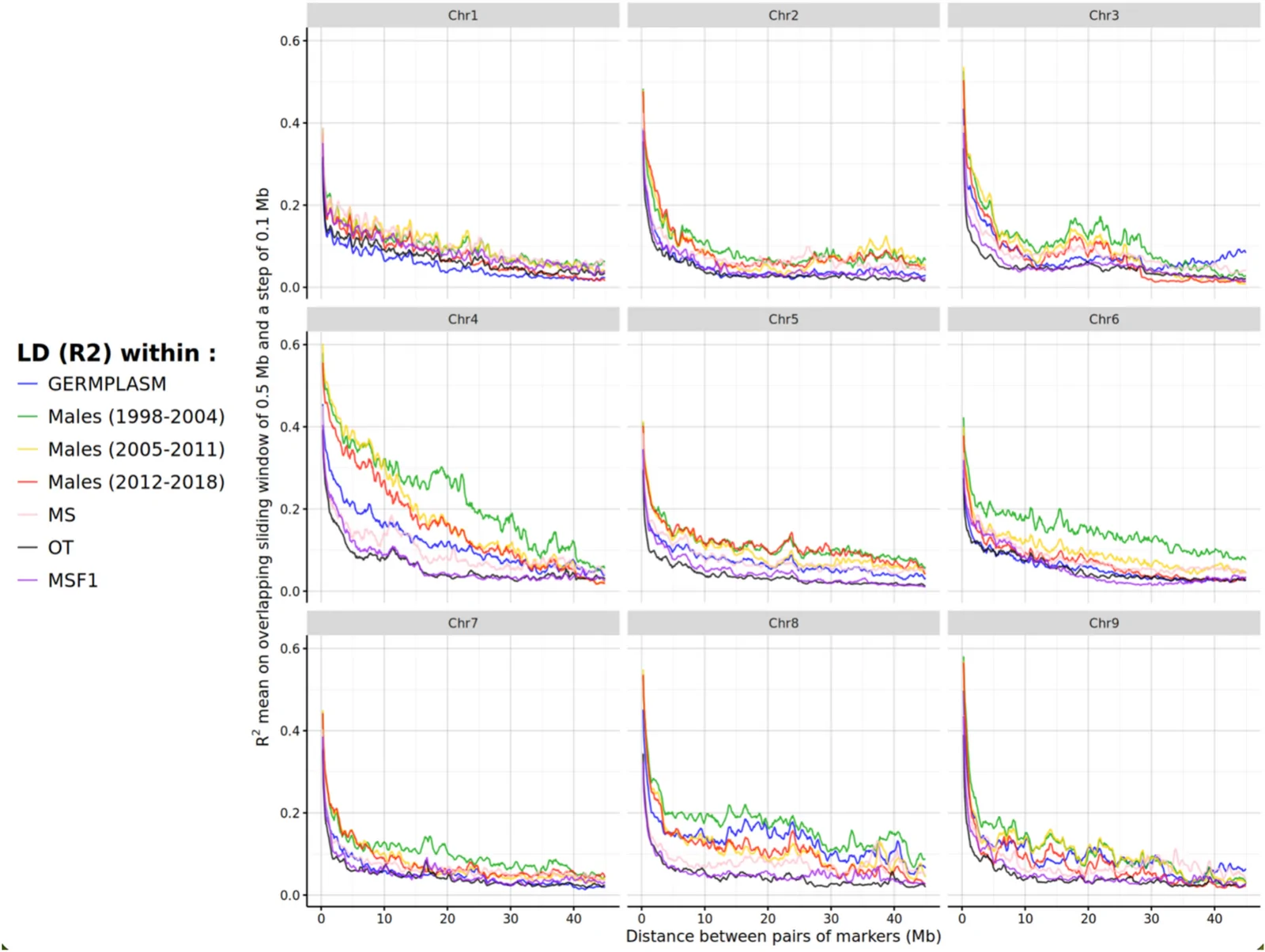
Genome-wide linkage disequilibrium (LD) decay within breeding components and over three eras within the male pool. GERMPLASM pool gathers all parental pools used to create haploblocks

For most chromosomes, LD decreased within the first 10 Mb of inter-marker distances before reaching a plateau of mean *r*^2^ values under 0.10. Despite this general pattern, the degree and rate of LD decay varied substantially among genetic pools, with the exception of chromosome 1 where LD showed a consistent pattern across parental pools (Fig. 6).

Short distance (< 1 Mb) LD was significantly lower in females than in males, and LD significantly decreased in males over time (*p*-values < 0.05). Among OT, 4.7% of marker pairs displayed *r*^2 ^> 0.25, whereas this proportion reached 8.84% in MS and 17.6% in males from the first breeding era (1998–2004). Note that the percentage of markers with *r*^2 ^> 0.25 decayed in males to 14.0% (2005–2011) and then 12.8% (2012–2018), corresponding to a mean *r*^2^ decrease of 25.2% in 21 years between all intra-chromosomic pairs of markers.

Chromosome 4 displayed remarkably strong LD, with a mean (over 0.5 Mb windows) *r*^2^ of 0.2 reached at 2.5 Mb for OT, 3.8 Mb for MS, 15.5 Mb for males obtained after 2005 and 22.8 Mb for males from 1998 to 2004. Older male lines (1998–2004, green) consistently showed high LD levels on most chromosomes, with pronounced peaks on chromosomes 3, 4, 6, 7, and 8, where elevated *r*^2^ values extended over longer distances. In contrast, more recent male lines (2012–2018, red) exhibited faster LD decay. LD in OT lines tended to be slightly lower than in MS lines, particularly on chromosomes 3, 4, 5, 6, 8 and 9 (Fig. 6), but the smaller MS sample size could influence the values. Because LD estimates can be sensitive to sample size, especially in smaller groups, comparisons involving the MS pool were interpreted cautiously.

LD was used to define haploblocks in order to assess haplotypic diversity while integrating allelic variation and effective recombination events. Parameter values for haploblock construction were based on an initial assessment. A D’ threshold of 0.2 was selected to balance the number of haplotypes per haploblock (on average 20 haplotypes per haploblock) and the size of the haploblocks (on average 24 SNPs and 1.3 Mb). Resulting haploblocks carried on average 2.7 effective haplotypes (HAe) within males, 3.6 within MS and 4.7 within OT. Within males, the effective number of haplotypes remained stable across breeding eras (HAe between first and third period: *p*.value = 0.3585).

### Putative selection targets between heterotic pools identified in seven large haploblocks

**Fig. 7:**
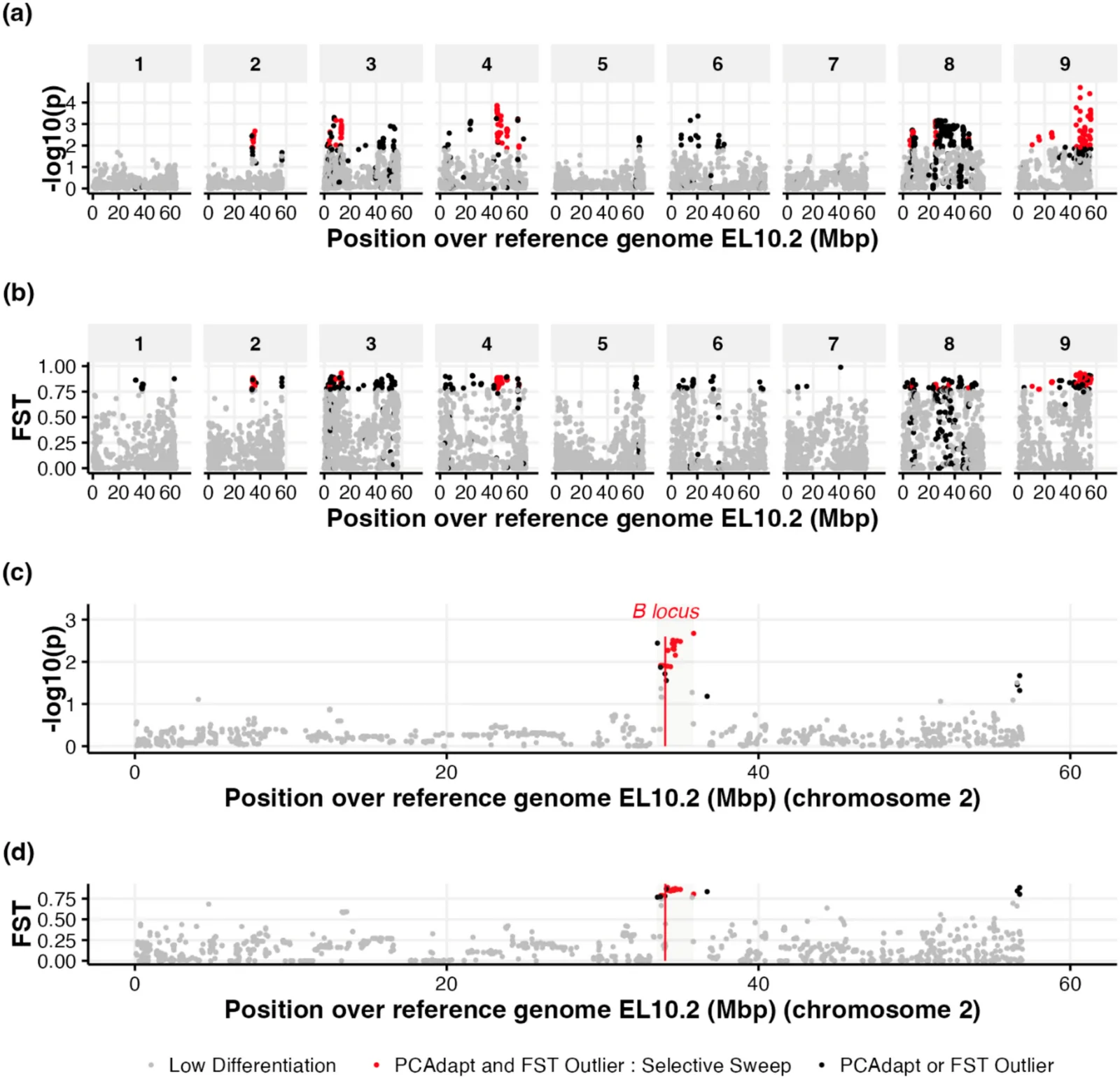
Selection signals associated with the differentiation between the male pool and MSF1. Black dots indicate outlier SNPs detected using one of the two statistics, and red dots those detected by both. (**a**), (**c**) PCAdapt -log(*p*-value) using first PCA axis (**b**), (**d**) Fst values between females and males. (**c**)–(**d**) zoom on chromosome 2 where outlier SNPs were detected in the B locus region (HAPLOBLOCK 1.1 and 1.2 in Supplementary Table S3)

To retrieve potential genomic targets of selection, we focused on comparisons between female hybrids (MSF1) and males and identified outlier SNPs displaying strong differentiation signals with both Fst and PCAdapt. We combined Fst outliers with PCAdapt outliers, using for the latter a significance threshold corresponding to a 10% FDR, representing the expected upper bound on the proportion of false positives among the loci identified as outliers. We identified 296 Fst outliers, 258 PCAdapt outliers, and 174 outliers common to both methods (Fig. 7). The 174 loci identified by both methods were located on five of the nine chromosomes, with most forming clusters that defined seven major haploblocks containing 100 of the 174 outlier SNPs (Supplementary Table S3).

Noteworthy, the chromosome 2 contained a cluster of outlier SNPs in a region spanning from 33,516 to 35,838 kb containing 50 annotated genes, among which the well-known bolting B locus that harbors nine genes including *BvBTC1* (Pin et al. 2012; McGrath et al. 2023) at position 34,012,098 (Fig. 7c, d). This cluster of outlier SNPs covered two haploblocks (Supplementary Table S3, Haploblocks 1.1 and 1.2). On chromosome 3, a 1.1 Mb selection signal (Haploblocks 2.2, Supplementary Table S3) covered the rhizomania-resistance region containing resistance gene analogs co-segregating with Rz1 (Gidner et al. 2005; Lein et al. 2007). While these overlaps support the biological relevance of the candidate regions, their causal effects on phenotypic variation remain to be evaluated. Note that the seven haploblocks that gather most of the outlier SNPs contained several genes with annotations related to flowering, disease resistance or stress responses (Supplementary Table S3).

To refine our understanding of selection patterns, we identified SNPs showing a shift in allele frequency in males between the first and second breeding era, the second and the third and the first and the third using the Fc statistic. Distribution of Fc-values was fitted with a beta distribution to identify loci with extreme Fc-values compared to the genome-wide Fc using a false positive rate of 5% (Supplementary Figure S6). Markers with a significant Fc-value showed an average absolute allele frequency shift of 0.43, compared to 0.10 for the remaining markers (Supplementary Figure S7). Among the 701 SNPs showing temporal dynamics, only 18 SNPs (3%) exhibited significant frequency changes across all intervals between the three periods, with a mean variation of 0.53. A slightly larger proportion of SNPs—329 SNPs (47%)—displayed gradual changes, characterized by a significant shift between the first and third periods (mean change: 0.40) but no significant intermediate shift. In contrast, 236 (34%) and 118 (17%) fell into a fast-changing category, displaying allele frequency shifts during the first or second interval, respectively (interval mean frequency variation: 0.45). Over the 21 years, 311 SNPs (88%) in the fast-changing category experienced a switch in their major allele, compared with only 132 SNPs (40%) in the gradual-changing category. Noteworthy, 314 SNPs had significant variations between at least two consecutive eras but followed non-monotonic trajectories, resulting in no overall significant shift.

Among the 701 temporal outliers, a majority (60%) also showed increased Fst between males and MSF1. Yet, among the 174 outlier SNPs differentiating males and female hybrids, only 18 (10%) were also detected as fast-changing allelic frequencies. This limited overlap between the two outlier categories indicates that they captured complementary signals: the former reflects genetic differentiation between males and females across the entire dataset, whereas the latter highlights allele frequency changes specific to male breeding eras. Among the genomic regions that exhibited both signals, the haploblock 3 on chromosome 4 (Supplementary Table S3) showed a marked temporal shift in males, with the most frequent haplotype frequency increasing from 37% in the first era (1998–2004) to 64% in the second era (2005–2011) and 85% in the third era (2012–2018). In parallel, the second most frequent haplotype declined from 45 to 25% and to 8% in males. Over the same time periods, the mean Fst across this 45-SNPs block rose from 0.69 to 0.76 between males and MSF1.

**Fig. 8:**
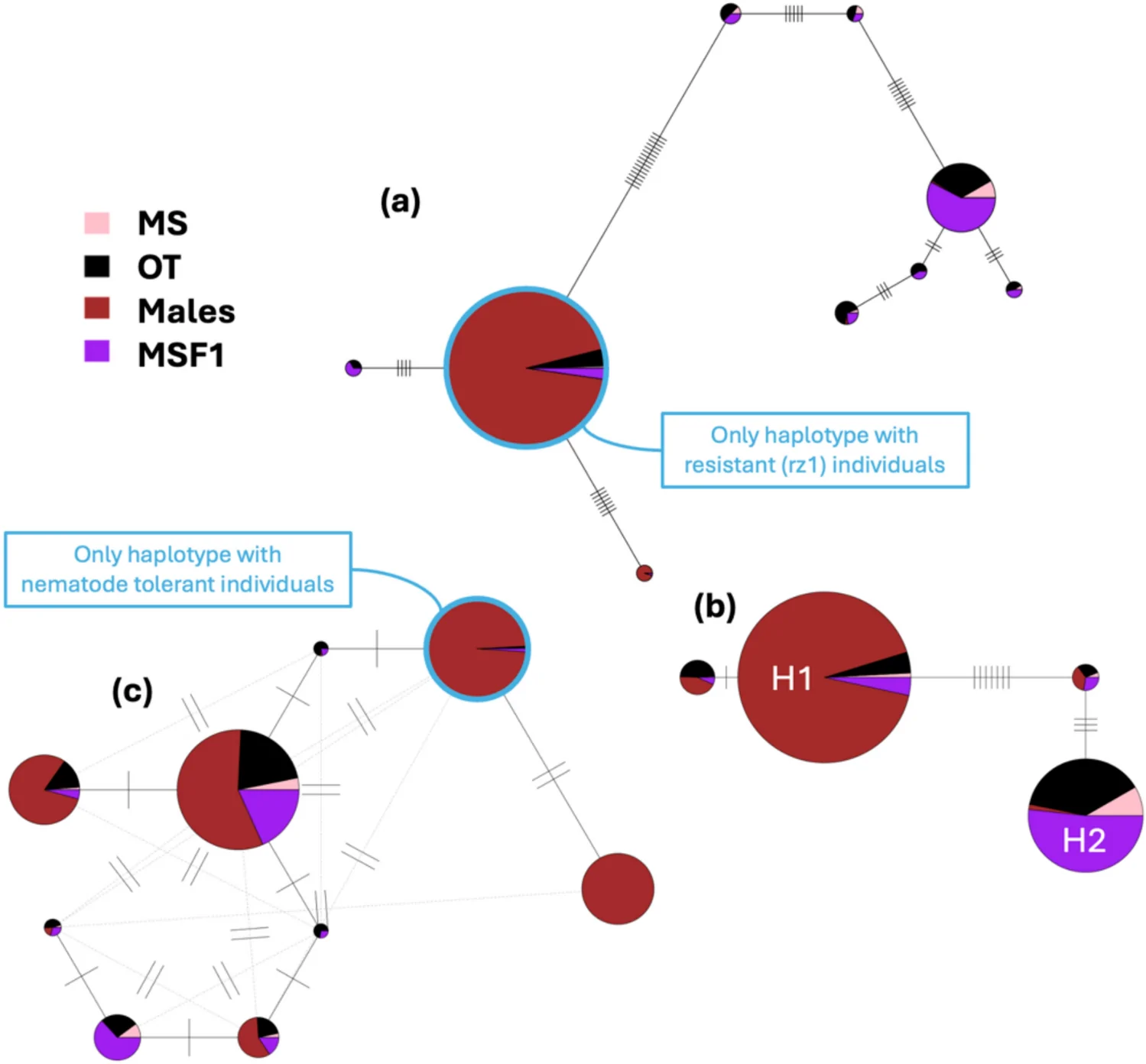
Haplotype network of (**a**) the 32 SNPs-wide haploblock 2.2 containing *RZ1* BNYVV resistance spanning 1.1 Mb, (**b**) the 20 SNPs-wide haploblock 1.1 containing *BvBTC1* spanning 690 kb and (**c**) the 5 SNPs-wide haploblock covering the BCN tolerance locus spanning 1.8 Mb. Haplotypes are filtered with frequency > 1%. Each node in the network is represented as a pie chart with color-coded segments proportional to the number of haplotypes found within pools and the length of edges proportional to the number of mutations (lines) separating nodes

We built a haplotype network encompassing 20 SNPs defining 15 distinct haplotypes within the *BvBTC1* haploblock (Haploblock 1.1, Supplementary Table S3) (Fig. 8b). Although the biennial allele is fixed in domesticated sugar beet, we observed an allelic series of 15 haplotypes (2.2 effective haplotypes within MS, 3.4 within OT and 1.1 within males). Interestingly, except for one haplotype, all other haplotypes associated distinctively with males or females (Fig. 8a). Haplotype H1 was carried by 93% of males but only 22% of MS and 17% of OT, while H2 was found in 63% of MS, 47% of OT and only 0.4% of males. The number of allelic differences between the two most frequent haplotypes (H1 and H2) was 10 SNPs, representing half of the total haploblock length. A similar haplotypic pattern appeared on the haploblock 2.2 comprising *Rz1* with two major haplotypes (including > 90% of male accessions and > 50% of female accessions, Fig. 8a). In contrast, we did not detect selection signatures at any of the 5 SNPs which define the haploblock spanning the BCN tolerance locus on chromosome 5 that has been recently introduced in the breeding pool (Stevanato et al. 2015; Sielemann et al. 2023). However, the haplotypic structure uncovered a diversity of haplotypes at high frequency (9 haplotypes > 1%: 217 observations) with a specific BCN tolerance haplotype found almost exclusively in males (> 95%) (Fig. 8c). This haplotype was carried by 9% of males of the 1998–2004 period and by 43% of males of the 2012–2018 period, a major haplotype frequency variation while only two SNPs of the dataset vary. No specific SNP was linked to this haplotype, which explains the lack of signal regarding frequency variation in this five-SNPs haploblock.

## Discussion

Plant breeders manage populations to improve a wide range of traits by selecting favorable alleles and allelic combinations while conserving genetic diversity to maintain long-term breeding potential. Breeding strategies have twofold consequences: (i) progressive directional selection, which leads to allele fixation in regions whose size depends on selection intensity and recombination events**,** and (ii) the recurrent use of new founders with specific features, resulting in bottlenecks and haplotype fixation which together impact diversity (Bevan et al. 2017; Allier et al. 2019). In hybrid breeding—whether two-way or three-way—maintaining and utilizing distinct heterotic parental gene pools is fundamental to fully exploit heterosis. Cytoplasmic male sterility is used to form the female gene pool in most hybrid crops, except maize, which benefits from mechanical detasseling. While two-way hybrids combine two heterotic parents, three-way hybrids introduce a third parental line in the female pool to reduce inbreeding depression and increase hybrid seed yield (Vendelbo et al. 2020; Collinson et al. 2022). Three-way hybrid breeding has been used in maize, rice, sorghum, rye, and sugar beet, though only rye and sugar beet breeders use it exclusively. Focusing on sugar beet three-way hybrid breeding, we present the first comprehensive analysis of a European elite proprietary germplasm, comprising 1285 accessions developed over 21 years of breeding and genotyped at 10k SNP markers, to evaluate how selection and diversity have been managed within and between heterotic pools. The use of a SNP array introduces an ascertainment bias because markers are selected from a discovery panel (Nielsen et al. 2004; Albrechtsen et al. 2010). This bias likely contributes to the deficit of rare alleles observed in the allele frequency spectra (Fig. 3). However, because these markers were designed based on sequencing data of accessions covering a broad diversity across the *Beta* genus not limited to the elite male and female pools (Mangin et al. 2015; Pegot-Espagnet et al. 2019; Huyghe et al. 2020; Dhiman et al. 2025) and because all pools and breeding eras were analyzed using the same set of markers, relative comparisons among pools and temporal periods are informative.

Genetic structure analysis clearly separated the two parental pools, suggesting the presence of two gene pools with a limited degree of recent admixture corresponding to advanced hybrid breeding patterns. A similar structure was reported in a proprietary rye germplasm derived from three-way hybrid breeding (Duvick 1959; Vendelbo et al. 2020). In rye, differentiation between female hybrids and males, measured by Fst, ranged from 0.23 to 0.33 (Bauer et al. 2017). In maize, Kadoumi et al. (Kadoumi et al. 2025) reported a mean Fst of 0.21 between two of the main heterotic pools used in modern breeding. These values are comparable to the Fst of 0.26 observed in our study. Although differentiation between heterotic pools is expected in hybrid breeding systems and likely contributes to heterosis through complementarity between parental pools (Comstock et al. 1949; Reif et al. 2005; Labroo et al. 2021), our study does not directly test this relationship. Our analyses are based solely on genomic data, so we cannot determine whether the observed genomic complementarity translates into functional complementarity, as reflected by hybrid performances or combining abilities.

Sugar beet breeding is inherently asymmetrical between male and female pools (Figs. 1, 2). The introduction of CMS into female accessions requires six to seven generations which slows down the selection process, whereas male accessions can be managed more efficiently, resulting in an unbalanced dataset with fewer female than male accessions. Hence, temporal analyses could only be reliably performed for the male pool. For this pool, we could identify three breeding eras, while the female pool lacked temporal resolution. Temporal changes in Fst should therefore be interpreted as changes in the male pool relative to the sampled MSF1 reference, rather than an analysis of simultaneous divergence between the two parental pools. In other hybrid crops, reciprocal recurrent selection contributes to the differentiation between parental pools (Feng et al. 2006; Gerke et al. 2015) and gradually reduces the contribution of SCA relative to GCA (Reif et al. 2005). In sugar beet, this differentiation occurs in an asymmetrical breeding system in which the two parental pools are managed under partly distinct constraints: the female pool is shaped by CMS maintenance, monogermity, seed-parent fertility and seed production, whereas the male pool is selected as the pollinator component of three-way hybrids evaluated mostly on root-related traits. It is important to note that, beyond the effects of selection, genomic differentiation may arise from several non-mutually exclusive processes, including genetic drift, which can be particularly pronounced in breeding populations with small effective population sizes, as well as founder effects and the targeted introgression of resistance sources (Li et al. 2011; Wang et al. 2016).

The asymmetry of selection schemes between male and female pools likely also contributes to differences in LD patterns. The lower LD observed in females compared with males, in contrast to what has been reported in rye for example (Adetunji et al. 2014; Vendelbo et al. 2020), may reflect differences in breeding history and selection constraints between the two pools. In particular, the lower LD in the female pool could result from less intense selection compared with the male pool. However, comparisons of LD among pools should also be interpreted in light of differences in sample size. Smaller groups, particularly the MS pool, may lead to less precise estimates of pairwise LD (Flint-Garcia et al. 2003). This limitation is unlikely to fully explain the temporal decrease in LD observed within males, as this trend was assessed across relatively large male subsets, but it may affect direct comparisons of LD among MS, OT, and male pools.

Interestingly, the structure within the male pool revealed a temporal pattern, identifying three distinct breeding eras in the 21 years covered by our study as inferred from admixture (*K *= 6, Fig. 2). Although these eras were defined retrospectively and do not reflect intentional changes in breeding practices, limiting their biological interpretation, they provide a rare opportunity to investigate temporal dynamic of differentiation. This temporal perspective is particularly valuable because it helps disentangle the effects of genetic drift from directional selection (Gerke et al. 2015; Buffalo and Coop 2019). Whereas drift generates stochastic changes in allele frequencies, directional selection produces consistent temporal shifts across breeding eras (Buffalo and Coop 2019). Using longitudinal data, we uncovered a strong effect of breeding on allele frequency. On one hand, the group of 18 SNPs exhibiting significant frequency changes across all time intervals were associated with the replacement of specific haplotypes by others and represent promising candidate regions for investigating genes underlying agronomically important traits. On the other hand, the group of 329 SNPs exhibiting more gradual changes between the first and third period are more likely to have been subject to weak selection. Finally, the 314 SNPs showing non-monotonic, yet strong variation are more likely to reflect drift in the male pool.

Our analyses uncover two temporal layers of genomic differentiation: a long-term divergence between male and female pools, detected by PCAdapt and Fst outliers and predating the 21-year period examined here, and more recent selection within the male pool, captured through Fc and temporal changes in MRD and Fst. Interestingly, we retrieved the rhizomania-resistance region (Gidner et al. 2005; Lein et al. 2007) and the well-known bolting B locus (Pin et al. 2012; McGrath et al. 2023). Several other genes linked with flowering and diseases resistances overlap around differentiation signals, however, their effects need to be experimentally validated. The BNYVV resistance locus *Rz1* (Scholten and Lange 2000; Gidner et al. 2005) encompasses a dominant allele conferring tolerance against root viruses of the genus *Benyvirus*. This gene has been introduced in breeding germplasms after its appearance in *Rizor* and *Holly* varieties after 1984. *Rz1* is now widely used in commercial hybrids, often combined with *Rz2* (Benjes et al. 2024). Differentiation scan combined with haplotype analysis around the BCN tolerance locus (Fig. 8c) highlights the increase of a specific haplotype frequency within males (from 9% in the 1998–2004 period to 43% in the 2012–2018 period). Haplotypic pattern matches an introduction of the BCN tolerance in the male pool in the early 2000s from wild relatives (Lewellen 2006; Stevanato et al. 2015; Sielemann et al. 2023). However, this region was not detected as a selection footprint. This may reflect the recent selection for BCN tolerance and/or the absence of SNPs tightly linked to causal variants, so that the signal is detectable at the haplotype level but not at the level of individual SNPs. We found selection signals at the bolting locus. Among the nine genes clustered at this locus, *BvBTC1* has been identified as the major gene underlying the annual and biennial growth habit. The two alleles differ by 11 nonsynonymous SNPs and a large insertion of approximately 28 kb in the promoter region that is unique to the biennial allele. Previous studies suggested that the biennial allele became fixed in sugar beet following domestication (Abegg 1936; Pin et al. 2012; Frerichmann et al. 2013; Höft et al. 2018). Surprisingly, we uncovered in our biennial elite germplasm an allelic series at the B locus and a differentiation between males and females. The haplotypic differentiation reported here most likely reflects the contribution of multiple founder haplotypes to the elite germplasm consistent with selection on pre-existing standing genetic variation at this locus during domestication (Stitzer and Ross‐Ibarra 2018). Phenotyping will be needed to determine whether these haplotypes have functional consequences. Haploblock 3 on chromosome 4 shows persistent divergence between the male pool and the female hybrid pool, together with directional selection in the former, where one haplotype has been consistently favored over the past 21 years. This region therefore represents a promising candidate for future studies investigating breeding-driven haplotype selection and variation at phenotypic traits.

## Conclusion

This study reveals that 60 years of three-way hybrid breeding in sugar beet have generated measurable genomic divergence between male and female heterotic pools and that recent breeding carried out during the last 21 years increased this divergence while slowly reducing within-pool diversity. The combination of reciprocal selection, recombination, drift and founder effects has contributed to distinct haplotypic architectures between pools at agronomically relevant loci such as the B locus region containing BvBTC1 and the rhizomania-resistance region containing Rz1 (Gidner et al. 2005; Lein et al. 2007; Pin et al. 2012; Felkel et al. 2023). Future work integrating hybrid performance, combining ability and trait-specific phenotyping will be required to infer the phenotypic consequences of this differentiation and determine how it contributed to heterosis and long-term genetic gain.

## Supplementary Information

Below is the link to the electronic supplementary material.

Supplementary file1 (PDF 1533 KB)

Supplementary file2 (XLSX 728 KB)

## Data Availability

The data analyzed in this study are part of an active commercial sugar beet breeding program of United Beet Seeds and are composed of most of the key lines of its western Europe material, from 21 years old to current experimental selection lines. As such, the data, genotypic material and pedigree are confidential and protected as intellectual property or as trade secrets. Consequently, datasets are not publicly available. R code can be made available upon request, and principal analysis results are within the paper or its Supporting Information Files.
