# Supplementary material for: Twenty-one years of three-way hybrid breeding shaped diversity and complementarity of male and female gene pools in sugar beet
Source: Theor Appl Genet. 2026 Sep 19;139(10):271. doi: 10.1007/s00122-026-05366-8 (PMC13589703; doi:10.1007/s00122-026-05366-8)
Supplement: Supplementary file 1 [file 122_2026_5366_MOESM1_ESM.pdf]

Supplementary material for : Twenty-one years of three-way hybrid breeding shaped diversity and complementarity of male and female gene pools in sugar beet

DESPREZ Augustin, HENRY Karine, DESPREZ Bruno, DEVAUX Pierre, CHARCOSSET Alain, TENAILLON Maud & MOREAU Laurence

Supplementary Information : Genotyping data management

Off-Types Identification

Off-type individuals are plants assigned to a given accession but are genetically distinct from the accession. They typically arise from labeling, genotyping or crossing errors, or unintended pollination. If identified during selection of parental lines, off-types are discarded before contributing to progeny. Here we used genotyping data to identify off-types and remove them from downstream analyses.

To identify off-type plants within accessions, we measured genetic distances between plants of a given accession. Genotyping data were formatted numerically, with allelic dosages assigned as follows: 0 for homozygous AA, 1 for homozygous BB, and 0.5 for heterozygous AB. Each plant was represented as a vector of allelic dosages across l total number of genotyped markers.

The genetic distance used is the Manhattan distance δ, representing the sum of absolute differences across all l markers. The standardized Manhattan genetic distance between two genotypes, based on l markers, was computed as $\delta = \frac{\sum_{i=1}^{l} |p_{xi} - p_{yi}|}{l}$ where p_{xi} and p_{yi} are the allelic dosage vectors for individuals x and y at marker i. To enable interpretation relative to marker density, the Manhattan distance was standardized by dividing by l, yielding an average per-marker distance between individuals.

For off-type detection, we selected the Manhattan distance due to its linear properties and intuitive normalization. Within each accession, pairwise Manhattan distances were calculated between all plants separately for each chromosome. Mean Manhattan distances computed per plant within an accession followed a normal distribution. Therefore, we applied a Z-test (implemented in R package BSDA) to assess whether a plant's mean distance exceeded the expected variation within its accession, using a 95% confidence interval. We established a chromosome-specific threshold and classified a plant as an off-type when at least seven of its nine chromosomes were identified as divergent. This criterion was used to minimize the risk of discarding individuals with residual local heterozygosity.

Consensus genotype construction

Each accession consisted either of progeny obtained by self-fertilization of a single individual, corresponding to S2 or S3 lines for the OT, MS, and male accessions, or of several individuals representing the same hybrid accession, including MSF1 and three-way hybrids. Genotypic data from multiple plants (N) were therefore combined to assign a single consensus genotype to each accession at each biallelic SNP marker.

For a marker with alleles A and B, six possible accession-level genotypic profiles (Supplementary Table S1) were considered according to the expected distribution of genotypes among the sampled plants: [AA], corresponding to 100% AA individuals; [BB], corresponding to 100% BB individuals; [ABfix], corresponding to 100% AB individuals; [AAB], corresponding to 50% AA and 50% AB individuals; [ABB], corresponding to 50% AB and 50% BB individuals; and [ABseg], corresponding to 25% AA, 50% AB, and 25% BB individuals. All six profiles were assigned a likelihood considering the observed genotype frequency following a trinomial pattern in order to choose a consensus (Supplementary Table S2). Together, these six profiles encompass all expected offspring genotype distributions arising from self-fertilization or crossing when each parent is represented by a single diploid plant with genotype AA, AB, or BB. The parents of the studied male and female accessions were individual plants and could therefore carry only one of these three genotypes at a biallelic marker. In breeding programs, crosses are likewise generally performed between selected individual plants. Although commercial-scale seed production may involve crosses between multiple plants, parental accessions are maintained as highly homogeneous and genetically stable lines. Each parental group can therefore be approximated as carrying a single genotype at a given marker, making the resulting offspring distribution statistically comparable to that expected from a cross between two individual parental plants.

Table S1: Possible genotypic profiles of accessions used to assess a consensus genotype.

Genotype of the accession's parents	**AA**	**BB**	**AB**
**AA**	100% AA<br>genotypic profile **[AA]**	100% AB<br>genotypic profile **[ABfix]**	50% AA<br>50%AB<br>genotypic profile **[AAB]**
**BB**	100% AB<br>genotypic profile **[ABfix]**	100% BB<br>genotypic profile **[BB]**	50% BB<br>50% AB<br>genotypic profile **[ABB]**
**AB**	50% AA<br>50% AB<br>genotypic profile **[AAB]**	50% BB<br>50% AB<br>genotypic profile **[ABB]**	25% AA<br>25% BB<br>50% AB<br>genotypic profile **[ABseg]**

Table S2: Likelihoods of possible genotypic profiles computed from genotype counts. Genotypic profiles are defined from parental genotypes. Likelihoods are computed from genotype counts (nAA, nAB, nBB) for a total of N observations and, considering an error rate, R. Errors are considered independent.

Genotypic profile of accession	Accession's Parental genotype	Genotype frequency in accession	Likelihood of observing accession's genotyping data considering each profile, with an error rate R of observing the wrong allele (genotyping errors + mutations), taking into account the number of AA, AB and BB in the accession.
[AA]	AA<br>AA	100% AA	$L(\text{AA} \mid OBS) = \frac{(1-R)^{n_{AA}} \left(\frac{-1+\sqrt{1+4R}}{2}\right)^{n_{AB}} \left(\frac{-1+\sqrt{1+4R}}{2}\right)^{2^{n_{BB}}} N!}{n_{AA}!\ n_{AB}!\ n_{BB}!}$
BB	BB<br>BB	100% BB	$L(BB \mid OBS) = \frac{\left(\frac{-1+\sqrt{1+4R}}{2}\right)^{2^{n_{AA}}} \left(\frac{-1+\sqrt{1+4R}}{2}\right)^{n_{AB}} (1-R)^{n_{BB}} N!}{n_{AA}!\ n_{AB}!\ n_{BB}!}$
ABseg	AB<br>AB	25% AA<br>50% AB<br>25% BB	$L(ABseg \mid OBS) = \frac{(\frac{1}{2})^{n_{AA}} (\frac{1}{4})^{n_{AB}} (\frac{1}{2})^{n_{BB}} . N!}{nAA!\ nBB!\ nAB!}$
ABfix	AA<br>BB	100% AB	$L(ABfix \mid OBS) = \frac{\left(\frac{R}{2}\right)^{n_{AA}} (1-R)^{n_{AB}} \left(\frac{R}{2}\right)^{n_{BB}} N!}{n_{AA}!\ n_{AB}!\ n_{BB}!}$
AAB	AA<br>AB	50% AA<br>50% AB	$L(AAB \mid OBS) = \frac{(\frac{1}{2} - (\frac{-1+\sqrt{1+2R}}{2})^2)^{n_{AA}} (\frac{1}{2} - \frac{-1+\sqrt{1+2R}}{2})^{n_{AB}} (\left(\frac{-1+\sqrt{1+2R}}{2}\right)^2 + \frac{-1+\sqrt{1+2R}}{2})^{n_{BB}} N!}{n_{AA}!\, n_{AB}!\, n_{BB}!}$
ABB	BB<br>AB	50% BB<br>50% AB	$L(ABB \mid OBS) = \frac{(\left(\frac{-1+\sqrt{1+2R}}{2}\right)^2 + \frac{-1+\sqrt{1+2R}}{2}\frac{1}{2})^{n_{AA}} (\frac{1}{2} - \frac{-1+\sqrt{1+2R}}{2})^{n_{AB}} (-(\frac{-1+\sqrt{1+2R}}{2})^2)^{n_{BB}} N!}{n_{AA}!\, n_{AB}!\, n_{BB}!}$

Three examples of consensus genotype inference based on progeny genotype counts.

In case 1, consider an accession genotyped at a given SNP for ten individuals, where nine are AA, one is AB and none is BB (Figure S1). The likelihood of each possible accession-level genotypic profile is computed using the observed counts and a genotyping error rate R = 0.04 (Supplementary Table 2). The resulting likelihoods for [AA], [BB], [ABfix], [AAB], [ABB], [ABseg] are 2.67×10^{-1}, 1.34×10^{-26}, 4.92×10^{-15}, 9.32×10^{-3}, 2.46×10^{-15} and 1.91×10^{-5} respectively (Figure S1). With Q=2, the likelihood of the most probable profile [AA] is more than Q that of the second most probable profile [AAB]. Therefore, the most likely profile was retained as the consensus genotype. In case 2, the presence of two AB individuals resulted in similar likelihoods for [AA] and [AAB] (4.8×10^{-2} and 4.0×10^{-2}, respectively). Because the likelihood of [AA] did not exceed the Q-fold threshold relative to the second most probable profile [AAB], no consensus was assigned and the accession was recorded as missing.

In case 3, for ten individuals, three are AA, five are AB and two are BB, a rather balanced distribution. The likelihood for [ABseg] is more than 23 times more probable than the second most likely profile ([ABB]). This result matches with the natural assignment considering [ABseg] being the only profile where all three genotype can be observed in the same accession, with observed frequencies far from errors expectations.

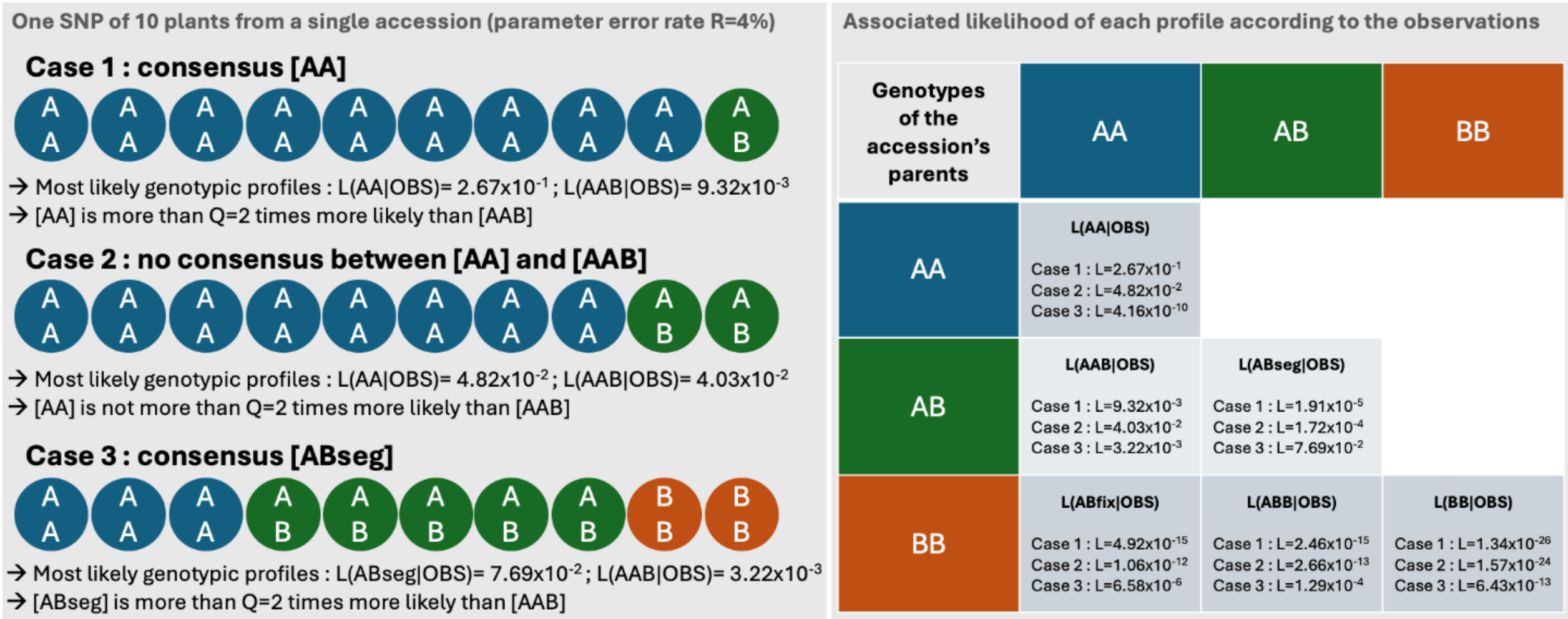

Figure S1: Three cases illustrating consensus genotype assignment. Observed genotype counts (left panel) allow to compute the likelihood of each genotypic profile (right panel) considering an error rate, R. The two most likely profiles are compared using a Q-fold likelihood threshold, and the most probable genotype was retained as the consensus genotype only when its likelihood was at least Q times higher than that of the second most probable profile. Parameter values are set to R = 0.04 and Q = 2.

Supplementary Figures

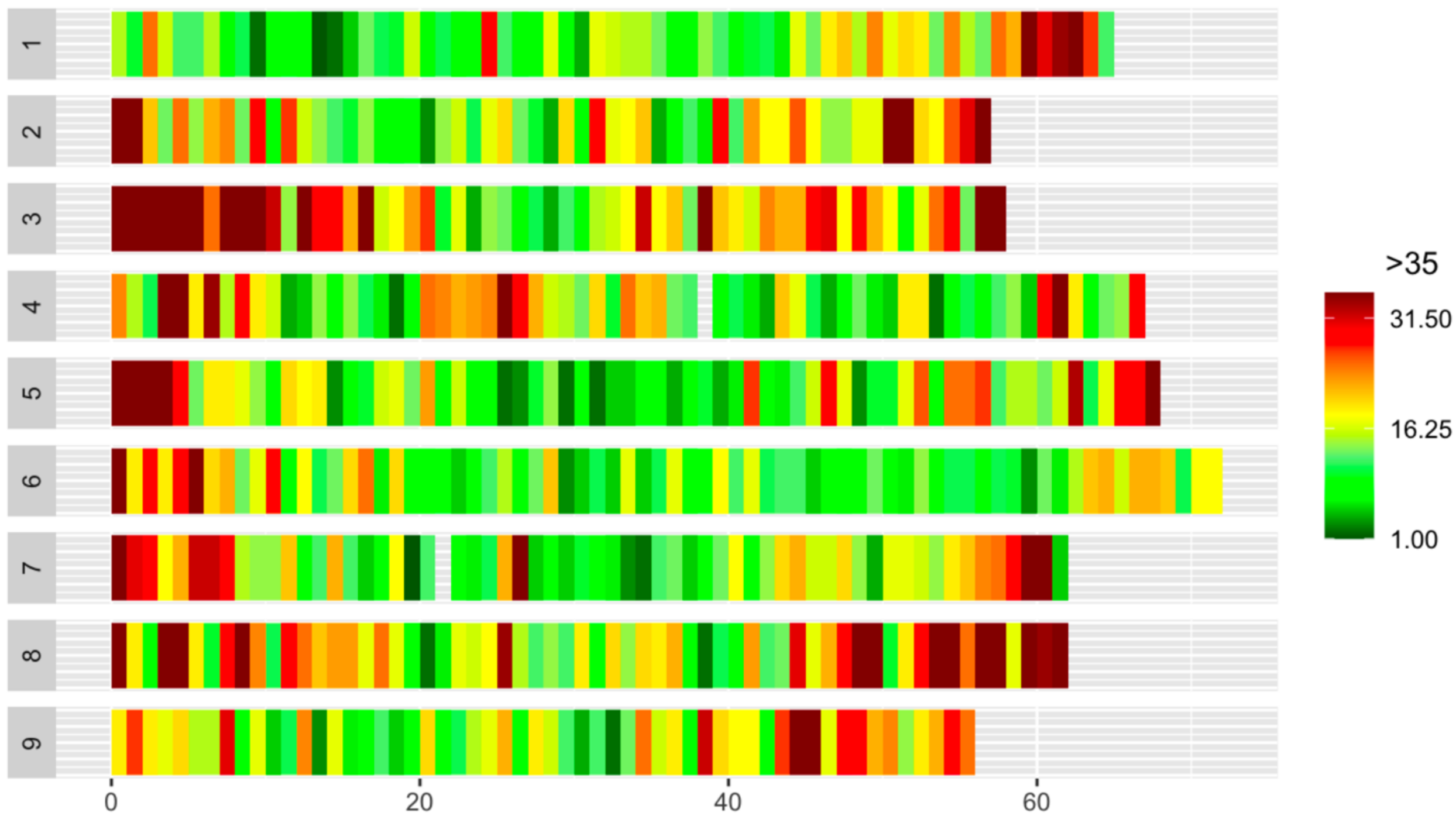

Figure S2: Marker density along the genome. Density is computed as the number of SNPs within 1Mb non-overlapping windows.

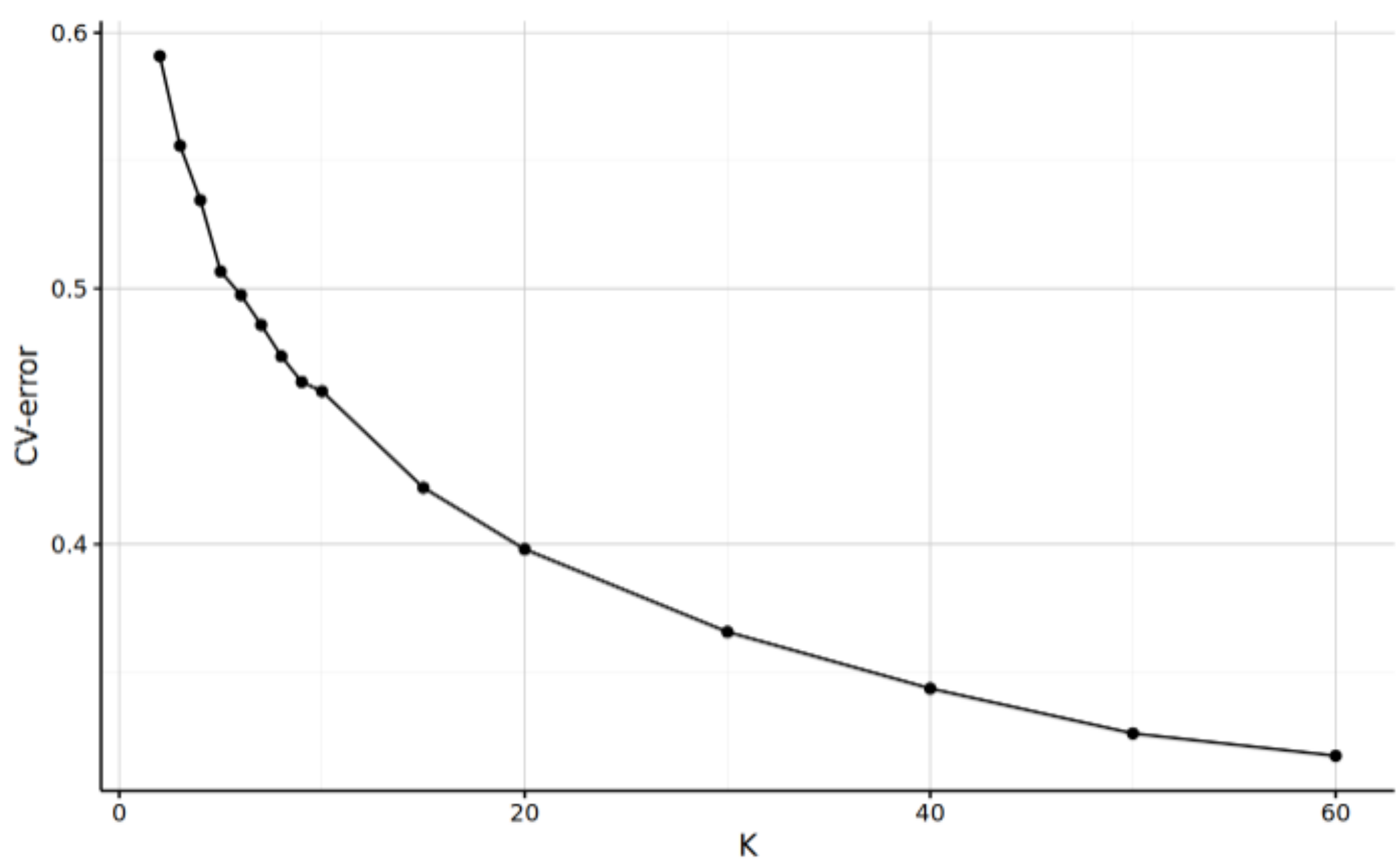

Figure S3: Cross-Validation errors from Admixture for a range of K values. K in {2,3,4,5,6,8,10,20,30,40,50,60}.

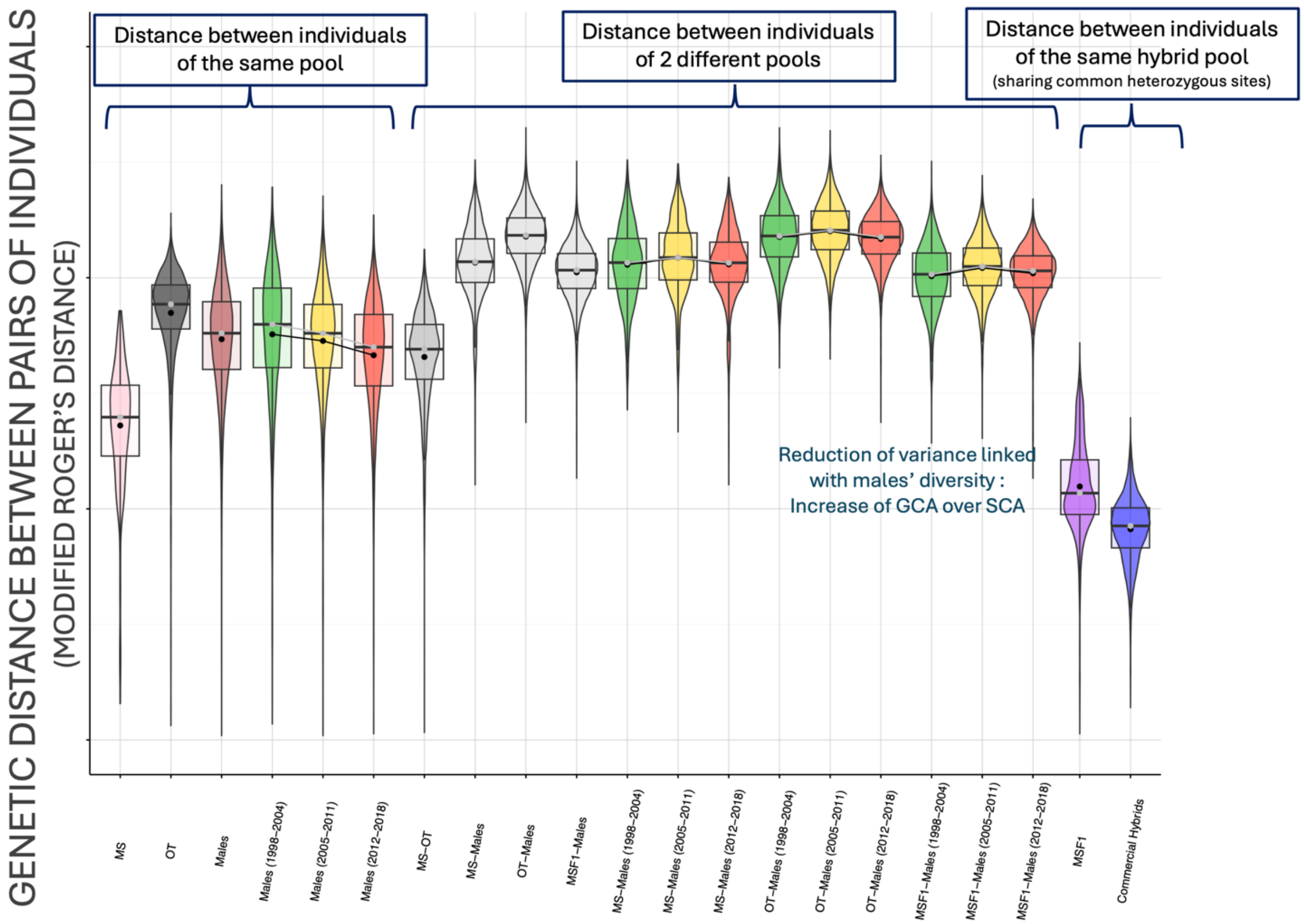

Figure S4: Violin plots of the genetic distance between individuals within and between gene pools. Genetic distance was computed as MRD within pools and between pools as indicated along the X-axis. Note that the male pool was separated in three eras (in green, yellow and orange).

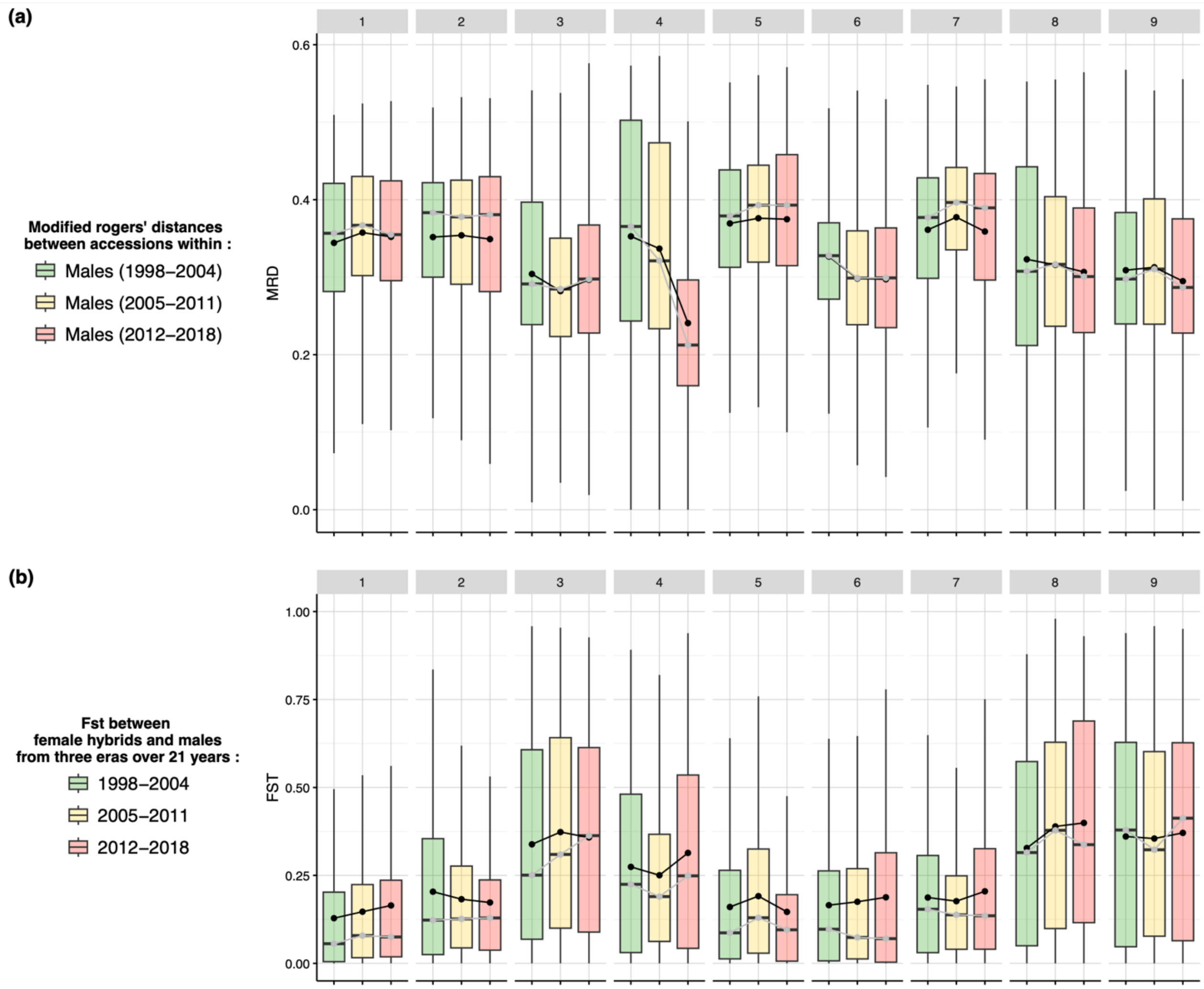

Figure S5: Box plots of the per-chromosome diversity and differentiation across three eras. Diversity within males (a) and differentiation between males and MSF1 (b) are shown across the three breeding eras. Median and mean values are shown in grey and black, respectively.

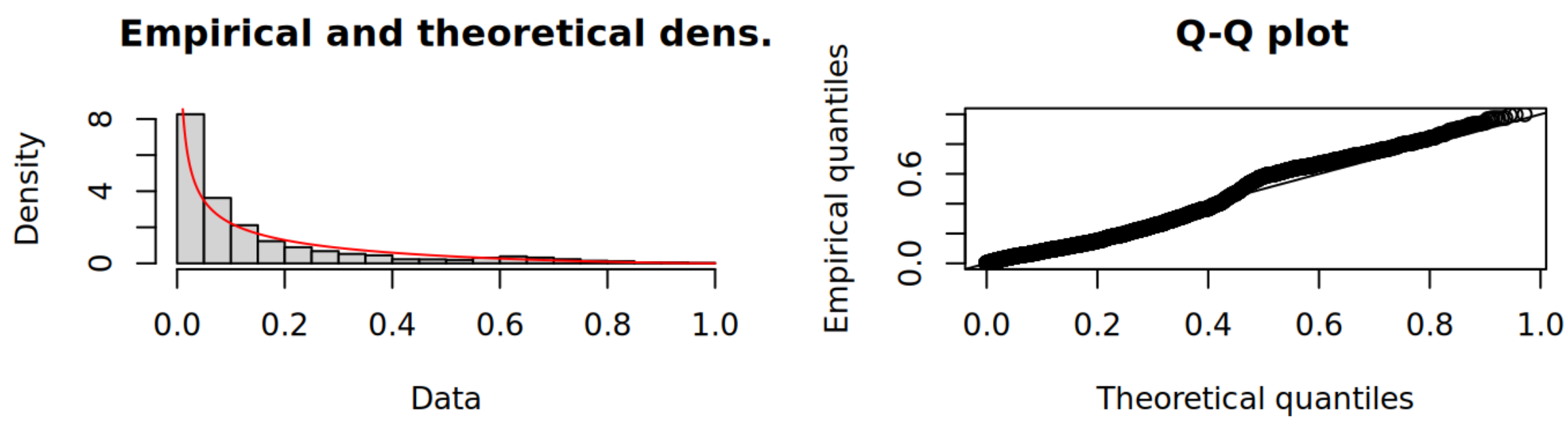

Figure S6: Distribution of Fc values and fitted beta distribution. The empirical distribution is fitted to a beta distribution in red (left) and both distributions are compared using a Q-Q plot (right).

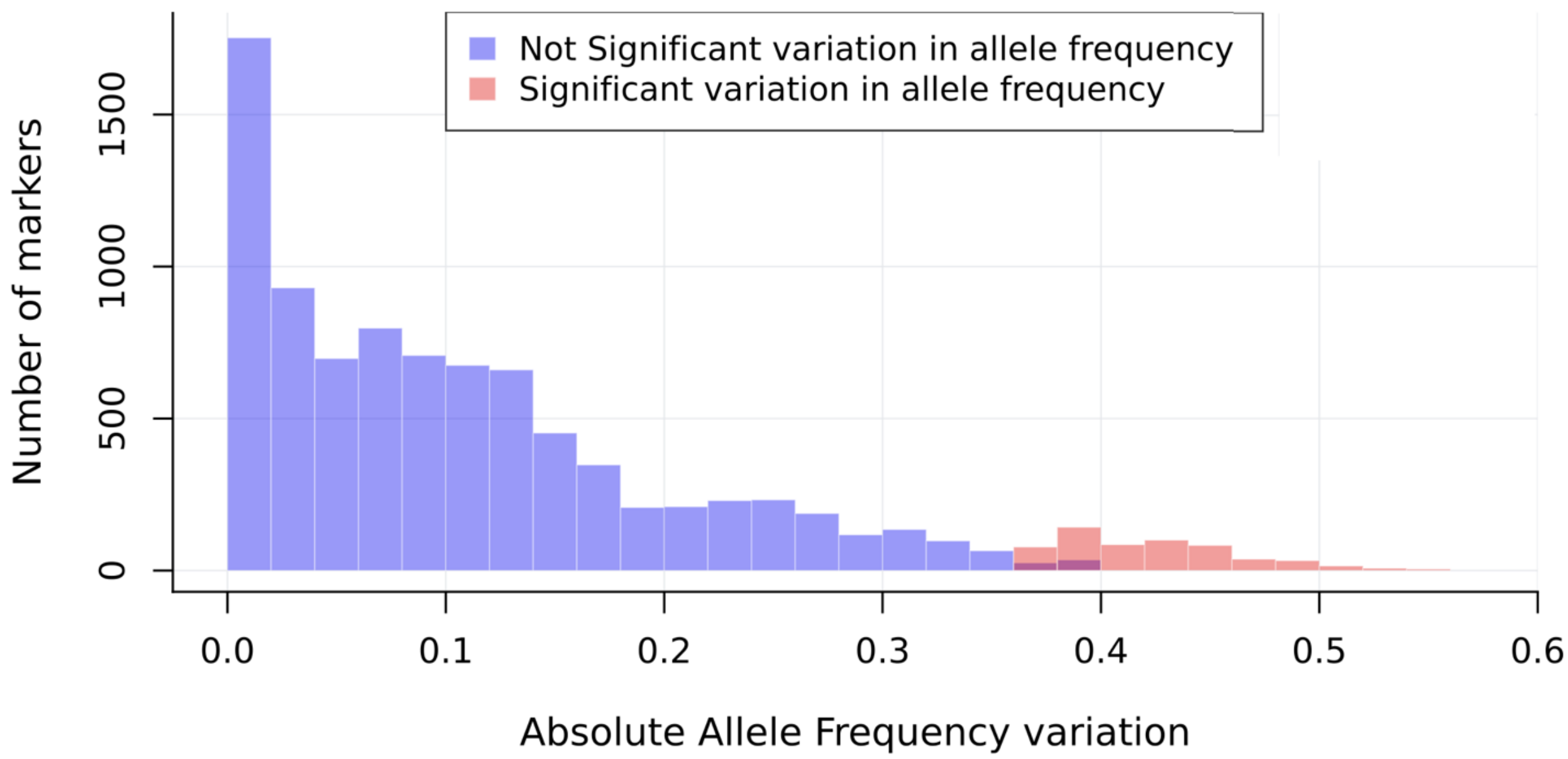

Figure S7: Allele frequency variations between the first and third breeding era according to their significance based on Fc. Significance threshold relies on a 5% FPR using a beta distribution over Fc (blue : no significant allelic frequency variation ; red : significant allelic frequency variation) .

Supplementary Table

Table S3: Identification of haploblocks associated with selection signals between heterotic pools and list of main candidate genes.

Haplo block	Chr	#SNP	Length kb	#Haplo types	#Annotated genes	Number of SNPs outliers for: Fst only	PCAdapt only	Fst & PCAdapt	Fc	Key genes/proteins
1.1	2	20	690	13	23	2	0	8	0	glutathione S-transferase T2-like, BvBTC1
1.2	2	10	1,570	13	46	0	0	6	0	salt stress-induced hydrophobic peptide ESI3-like, protein REBELOTE (flowering), 5*elongation factor 1-alpha
2.1	3	5	286	9	16	0	0	3	0	-
2.2	3	32	1,163	41	69	6	0	15	0	RGA2 RGA3 RGA4 (resistance gene analogues segregating with Rz1 (Lein et al. 2007)), FRS11 (unknown among FRS growth and flowering time genes)
3	4	45	4,490	61	187	1	2	31	2	sugar carrier protein C, flowering locus K homology domain, 11S, flowering-promoting factor, globulin seed storage protein, 2*disease resistance protein RPM1
4	8	40	1,165	52	80	9	7	14	0	FRS5 (regulating light control of development + BCTV resistance)
5.1	9	4	137	6	7	0	0	2	0	-
5.2	9	14	344	28	30	1	0	6	0	Mitochondrial linked genes (FMP32, CRM CFM9: growth and stress responses), FRS5 (regulating light control of development+ BCTV resistance)
6	9	20	760	45	79	2	0	6	0	F-box protein CPR1: early growth (cotyledon size) and disease resistance, FRS5 (light control of development + BCTV resistance) FRS11
7	9	19	255	19	20	1	0	13	0	8*glucosyltransferase proteins
